# Meiotic Crossover Control by Concerted Action of Rad51-Dmc1 in Homolog Template Bias and Robust Homeostatic Regulation

**DOI:** 10.1371/journal.pgen.1003978

**Published:** 2013-12-19

**Authors:** Jessica P. Lao, Veronica Cloud, Chu-Chun Huang, Jennifer Grubb, Drew Thacker, Chih-Ying Lee, Michael E. Dresser, Neil Hunter, Douglas K. Bishop

**Affiliations:** 1 Howard Hughes Medical Institute and Department of Microbiology & Molecular Genetics, University of California, Davis, Davis, California, United States of America; 2 Genetics Graduate Group, University of California, Davis, Davis, California, United States of America; 3 Committee on Genetics, University of Chicago, Cummings Life Science Center, Chicago, Illinois, United States of America; 4 Department of Radiation and Cellular Oncology, University of Chicago, Cummings Life Science Center, Chicago, Illinois, United States of America; 5 Weil Graduate School of Medical Sciences of Cornell University, New York, New York, United States of America; 6 Memorial Sloan-Kettering Cancer Center, New York, New York, United States of America; 7 Program in Cell Cycle and Cancer Biology, Oklahoma Medical Research Foundation, Oklahoma City, Oklahoma, United States of America; 8 Department of Cell Biology, University of Oklahoma Health Sciences Center, Oklahoma City, Oklahoma, United States of America; 9 Department of Molecular & Cellular Biology, University of California, Davis, Davis, California, United States of America; 10 Department of Cell Biology & Human Anatomy, University of California, Davis, Davis, California, United States of America; 11 Department of Molecular Genetics and Cell Biology, University of Chicago, Cummings Life Science Center, Chicago, Illinois, United States of America; National Cancer Institute, United States of America

## Abstract

During meiosis, repair of programmed DNA double-strand breaks (DSBs) by recombination promotes pairing of homologous chromosomes and their connection by crossovers. Two DNA strand-exchange proteins, Rad51 and Dmc1, are required for meiotic recombination in many organisms. Studies in budding yeast imply that Rad51 acts to regulate Dmc1's strand exchange activity, while its own exchange activity is inhibited. However, in a *dmc1* mutant, elimination of inhibitory factor, Hed1, activates Rad51's strand exchange activity and results in high levels of recombination without participation of Dmc1. Here we show that Rad51-mediated meiotic recombination is not subject to regulatory processes associated with high-fidelity chromosome segregation. These include homolog bias, a process that directs strand exchange between homologs rather than sister chromatids. Furthermore, activation of Rad51 does not effectively substitute for Dmc1's chromosome pairing activity, nor does it ensure formation of the obligate crossovers required for accurate homolog segregation. We further show that Dmc1's dominance in promoting strand exchange between homologs involves repression of Rad51's strand-exchange activity. This function of Dmc1 is independent of Hed1, but requires the meiotic kinase, Mek1. Hed1 makes a relatively minor contribution to homolog bias, but nonetheless this is important for normal morphogenesis of synaptonemal complexes and efficient crossing-over especially when DSB numbers are decreased. Super-resolution microscopy shows that Dmc1 also acts to organize discrete complexes of a Mek1 partner protein, Red1, into clusters along lateral elements of synaptonemal complexes; this activity may also contribute to homolog bias. Finally, we show that when interhomolog bias is defective, recombination is buffered by two feedback processes, one that increases the fraction of events that yields crossovers, and a second that we propose involves additional DSB formation in response to defective homolog interactions. Thus, robust crossover homeostasis is conferred by integrated regulation at initiation, strand-exchange and maturation steps of meiotic recombination.

## Author Summary

Meiosis is the specialized cell division that produces gametes by precisely reducing the chromosome copy number from two to one. Accurate segregation of homologous chromosome pairs requires they be connected by crossing-over, the precise breakage and exchange of chromosome arms that is carried out by a process called recombination. Recombination is regulated so each pair of homologous chromosomes becomes connected by at least one crossover. We studied the roles of two recombination proteins, Rad51 and Dmc1, which can act directly to join homologous DNA molecules. Our evidence supports the idea that Dmc1 is the dominant joining activity, while Rad51 acts indirectly with other proteins to support and regulate Dmc1. Furthermore, Hed1, an inhibitor of Rad51's DNA joining activity, is also shown to enhance the efficiency of crossing-over. Cells in which Rad51 is activated to promote DNA joining in place of Dmc1 have unregulated and inefficient crossing-over that often leaves chromosome pairs without the requisite crossover. Despite these defects, most cells that use Rad51 in place of Dmc1 complete meiosis and produce high levels of crossovers. Our results indicate that compensatory processes ensure that meiotic cells accumulate high levels of crossover intermediates before progressing to the first round of chromosome segregation.

## Introduction

During meiosis, haploid gametes are formed from diploid precursor cells via two successive rounds of chromosome segregation. By a program of events unique to meiosis, parental chromosomes (homologs) associate into homologous pairs and then disjoin from one another at the first division of meiosis (MI). In most organisms, the process of homologous recombination mediates both the pairing and disjunction of homologs [1]. Meiotic recombination initiates with the formation of numerous DNA double-strand breaks (DSBs; [2]). Nuclease processing of DSB-ends generates single-stranded tails, which then assemble into nucleoprotein filaments comprising RecA-family proteins, Rad51 and Dmc1, and their accessory factors [3], [4], [5]. These filaments mediate DNA homology search and strand invasion of a homologous template chromosome to form joint molecule (JM) intermediates [6], [7], [8]. In this way, recombinational interactions promote the pairing of homologs and their end-to-end connection by zipper-like structures called synaptonemal complexes (SCs; [9], [10], [11], [12]). A subset of recombination sites then form crossovers resulting in the stable interhomolog connections called chiasmata that facilitate homolog bi-orientation on the spindle and thereby promote accurate disjunction at meiosis I [13], [14].

The cell-to-cell variation in crossover numbers is much lower than the variation seen for DSB numbers [15]. This homeostatic regulation has been shown to buffer against stochastic and experimentally-induced variation of DSB numbers [16], [17], [18], [19], [20], [21]. Crossover homeostasis is inferred to reflect two key regulatory processes that define the upper and lower limits for crossover numbers [15]: (i) crossover assurance – each homolog pair obtains a minimum of one crossover, as required for accurate disjunction [22]; and (ii) crossover interference – adjacent crossovers are widely separated [23], [24], [25]. The mechanisms that underlie crossover homeostasis have not been defined, but potential for regulation at each step of meiotic recombination has been inferred. For example, at the initiation stage, DSB numbers appear to be modulated by signaling pathways involving the PI3-kinase-like kinases, ATM/Tel1 and ATR/Mec1 [26], [27], [28], [29], [30], [31]. During DNA strand exchange, template choice is biased to favor homologs over the sister chromatid, which is normally the preferred template for recombinational repair in mitotically cycling cells [32], [33], [34], [35]. Following initial strand exchange, crossing-over involves the formation of metastable JM intermediates, including double-Holliday junctions [36], while non-crossovers can arise from nascent D-loop intermediates [37], [38], [39], [40]. Finally, resolution of double-Holliday junctions is inferred to produce primarily crossovers, but alternative modes of resolution may also result in non-crossovers [40], [41], [42]. How regulation at each of these steps contributes to crossover homeostasis remains unclear.

In this study, specific relationships between Rad51, Dmc1, and their accessory factors are defined. Regulation of the strand-exchange step of meiotic recombination, especially template choice, has been a major focus of studies of meiotic recombination [32], [33], [43], [44], [45]. This includes understanding the functional relationships between the meiosis-specific RecA-homolog, Dmc1, and its counterpart, Rad51. Although both proteins are essential for meiosis, recent work has inferred that Rad51 does not participate directly in strand exchange, but facilitates Dmc1-mediated DNA strand-exchange [46]. In this study, specific relationships between Rad51, Dmc1, and their accessory factors are defined. Unexpectedly, Hed1, an inhibitor of Rad51's DNA strand exchange activity [47], [48], is shown to be required for efficient homolog template bias. Moreover, this function of Hed1 does not require the meiotic kinase, Mek1 [49], [50], [51]. Our analysis also reveals an unanticipated function for Dmc1 as an inhibitor of Rad51's strand exchange activity.

The *hed1* mutation also provides a means to test the inference that Rad51 can effectively substitute for Dmc1 during meiosis [47]. Contrary to this idea, severe defects in inter-homolog bias, homolog pairing, SC morphogenesis, and crossover assurance are observed in *dmc1 hed1* cells, where Rad51 catalyzes DNA strand exchange. We show that efficient crossover assurance requires homolog template bias and that even a mild reduction in interhomolog bias causes defective SC morphogenesis and renders cells sensitive to modest reductions in DSB numbers.

Remarkably, and despite the profound primary defects associated with Rad51-catalyzed meiotic recombination, crossing-over occurs at nearly wild-type frequencies. We show that most cells are able to compensate for defective interhomolog bias by biasing the outcome of recombination to favor crossing-over; and by a second feedback process that we propose involves additional DSB formation at sites that normally experience relatively low levels of recombination.

## Results

### Activation of Rad51 in the absence of Dmc1 allows repair of DSBs and progression of meiosis

In the budding yeast SK1 strain background, *dmc1* mutants arrest in meiotic prophase with unrepaired DSBs and profound defects in homolog pairing and SC formation [7], [52]. Previous studies have shown that activation of Rad51-mediated strand exchange allows *dmc1* mutants to progress through meiosis and form viable spores [10], [45], [47]. One interpretation of these observations is that Rad51 can effectively substitute for Dmc1 [10], [47].

**Figure 1:**
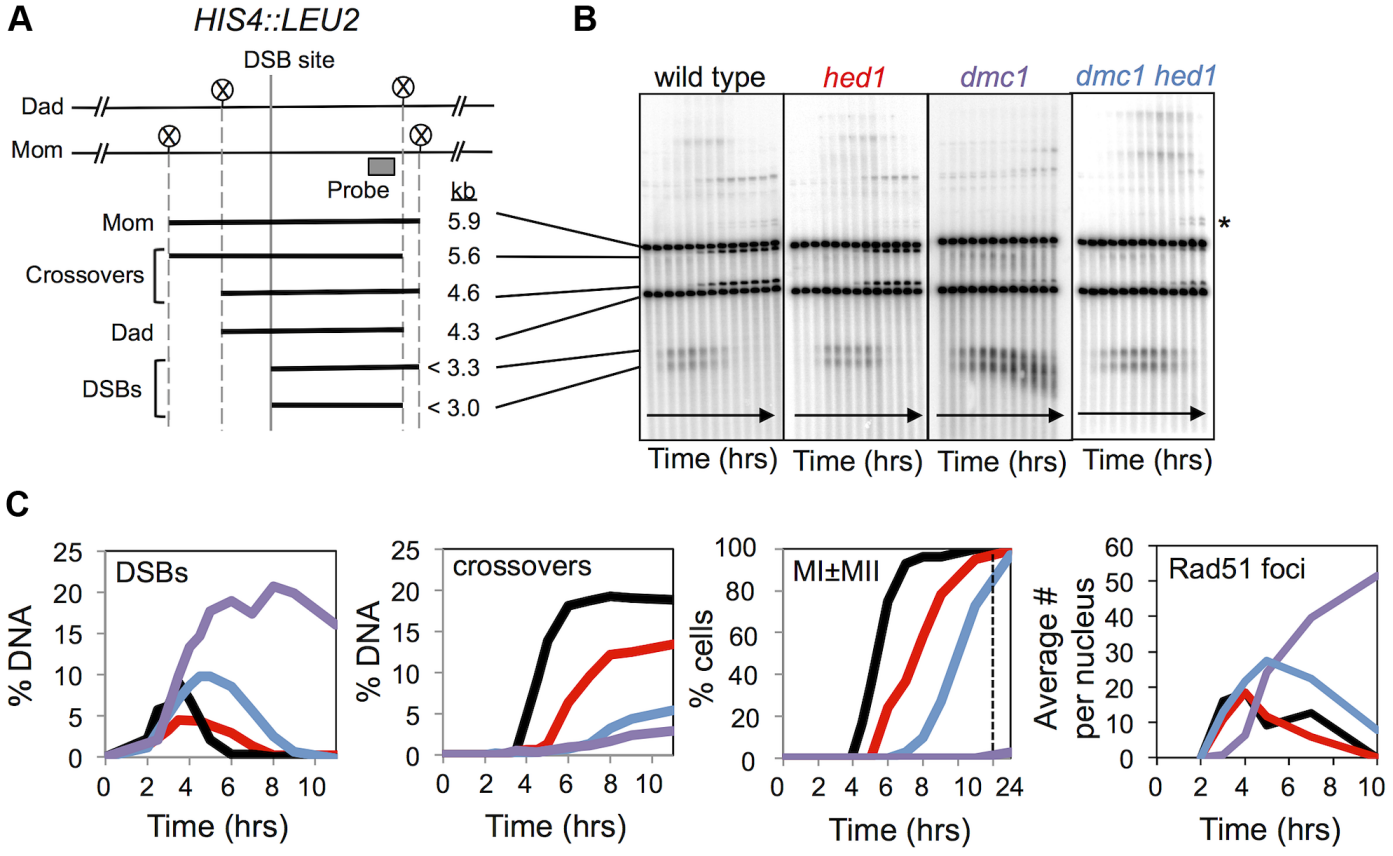
Analysis of DSBs and crossovers in *hed1* and *dmc1 hed1* mutants. A. Map of the *HIS4::LEU2* hotspot showing the DSB site, *Xho*I restriction sites (circled Xs) and the position of the probe used in Southern analysis. Sizes of diagnostic fragments are shown below. B. 1D Southern analysis of DSBs and crossovers at *HIS4::LEU2*. Time points are 0, 2, 3, 3.5, 4, 4.5, 5, 6, 7, 9, and 11 hours. Asterisk highlights bands diagnostic of non-allelic recombination between *HIS4::LEU2* and *leu2::hisG* located ∼25 kb away at the native *LEU2* locus. C. Quantitation of images shown in (B). % DNA is percentage of total hybridizing DNA signal. MI±MII is the percentage of cells that have completed one or both meiotic divisions. The rightmost panel shows quantification of Rad51 immuno-staining foci in spread nuclei. Each data point shows average Rad51 focus counts per nucleus (n = 50). The later data are from different time courses to those analyzed in the first three graphs.

To explore this possibility, the effects of activating Rad51's strand-exchange activity by deleting Hed1 were studied in detail at the *HIS4::LEU2* DSB hotspot in cell cultures undergoing synchronous meiosis (Figure 1, Figure S1; [53], [54], [55]). At *HIS4::LEU2*, restriction fragment polymorphisms between parental (Mom and Dad) chromosomes allow parental, DSB and crossover molecules to be resolved and monitored by Southern analysis (Figure 1A). DSBs appeared at the same time in cultures of wild type, *hed1*, *dmc1* and *hed1 dmc1* cells (Figure 1B,C). In wild type and *hed1* single mutants, DSBs peaked at 3.5 hours and were almost undetectable by 6 hours. As seen previously, DSBs formed in *dmc1* single mutants accumulated to very high levels and persisted for the duration of the experiment (Figure 1B,C). Abnormally extensive 5′-resection of DSB-ends was evident as a time-dependent increase in the heterogeneity and average mobility of DSB fragments (Figure 1B). Also consistent with previous work [10], [47], in the *dmc1 hed1* double mutant, DSB signals persisted much longer than in wild-type cells, but ultimately disappeared with a delay of about 2 hours.

Crossing-over within the 4.3 kb *Xho*I fragment surrounding the *HIS4::LEU2* DSB site plateaued at ∼19% of total DNA in wild-type cells (Figure 1A,B,C). Crossover formation was delayed in both the *hed1* single and *dmc1 hed1* double mutants relative to wild type. Furthermore, final crossover levels were reduced 1.4-fold in the *hed1* single mutant and 3.5-fold in the *hed1 dmc1* double mutant (see Figure S1E,F for additional measurements of final crossover levels).

MI was also delayed in *hed1* and, to a greater degree, *dmc1 hed1* cells. However, more than 90% of cells executed MI. To summarize, our analysis confirmed previous observations, which showed that meiotic DSBs are efficiently repaired in *dmc1 hed1* cells and can be converted into crossovers, albeit with significantly reduced efficiency [40]. Unexpectedly, absence of Hed1 alone also causes a significant decrease in crossing-over at the *HIS4::LEU2* recombination hotspot.

### Recombination template choice is severely defective in *dmc1 hed1* cells

**Figure 2:**
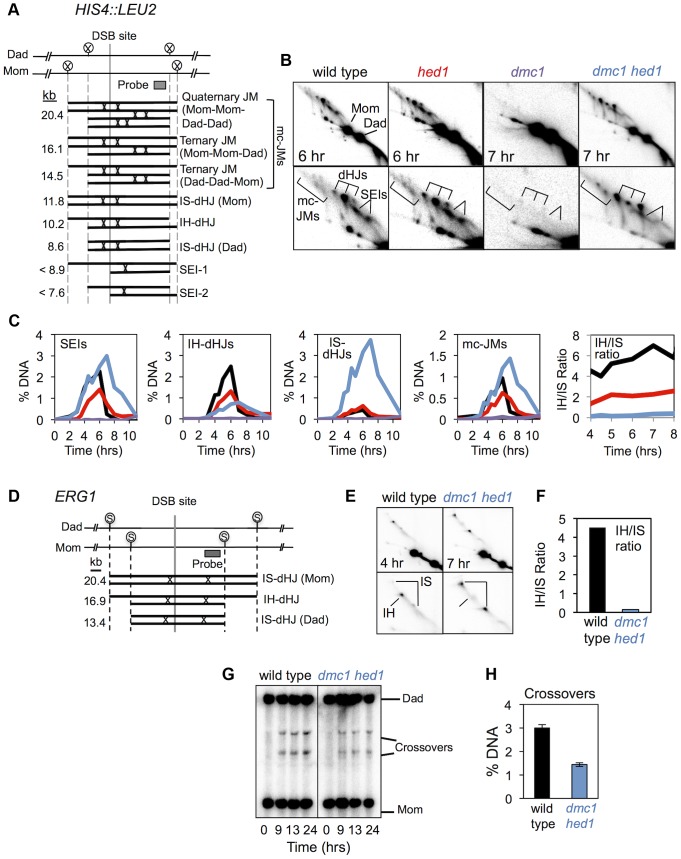
*hed1* and *dmc1 hed1* mutants are defective for interhomolog bias at two recombination hotspots. A. Diagram of JM structures detected at the *HIS4::LEU2* hotspot, from lowest to highest mobility. Positions of diagnostic *Xho*I sites (circled Xs) and the Southern probe are also highlighted. B. Images of 2D gels for time points that represent the peaks of JM abundance. Lower panels show magnifications of the JM signals. The positions of dHJs, SEIs, and mcJMs (multi-chromatid JMs) are indicated. C. Quantitation of JMs from 2D analysis. IH/IS ratio is the ratio of IH-dHJs/IS-dHJs. See Figure S1 for independent analysis of the same set of strains. D. Diagram of JM structures detected at the *ERG1* hotspot. Positions of *Sac*II sites (circled Ss) and the probe are shown. E. Images of 2D analysis at *ERG1* in wild type and *dmc1 hed1* strains. Positions of IH- and IS-dHJs are indicated. F. IH/IS dHJ ratios at *ERG1* in wild type and *dmc1 hed1* strains. G. 1D Southern analysis of crossing-over at *ERG1* in wild type and *dmc1 hed1* strains. H. Quantitation of images shown in (G). Crossover levels are shown for the 24 hr time point.

To examine the ability of Rad51 to substitute for Dmc1 in more detail, we analyzed JM formation in *dmc1 hed1* cells [47]. At the *HIS4::LEU2* locus, a variety of JM species can be monitored using 2D gels and Southern analysis (Figure 2A,B; [53], [54], [55]). These include single-end invasions (SEIs); double-Holliday junctions formed between homologous (IH-dHJs) or sister chromatids (IS-dHJs); and complex structures comprising three and four interconnected chromatids (ternary and quaternary multi-chromatid joint molecules, mcJMs).

Representative 2D gel images are shown in Figure 2B and quantitation of individual JM species is presented in Figure 2C. Consistent with previous data, JM formation is almost completely blocked in *dmc1* single mutants. Strikingly, *dmc1 hed1* cells form high levels of JMs, but the majority involve sister chromatids not homologs. Whereas wild-type cells form >5-fold more IH-dHJs than IS-dHJs, the IH-dHJ/IS-dHJ ratio is reduced from 5.7 to 0.22 in *dmc1 hed1* cells (Figure 2C; also see Figure S1). This 25-fold difference in the IH-dHJ/IS-dHJ ratio indicates that the processes responsible for interhomolog bias are severely defective in *dmc1 hed1* mutants.

To rule out the possibility that the inverted template bias seen in *dmc1 hed1* cells reflects differential formation and/or resolution of IH-dHJs relative to IS-dHJs, we also determined IH/IS ratios in the *ndt80* mutant background, in which JM resolution is blocked [40](Figure S2). JMs were analyzed at 7 and 8 hrs after induction of meiosis, when they have accumulated to maximum levels. As in *NDT80^+^* cells, the IH/IS dHJ ratio was 25-fold lower in *dmc1 hed1* cells than in wild type, when examined in the *ndt80* mutant background (Figure S2B). This result confirms that the low IH/IS dHJ ratio seen in *NDT80^+^ dmc1 hed1* cells results from defective template choice.

Additional changes in JM processing can also be discerned in *dmc1 hed1* mutants. Peak levels of both SEIs and mcJMs are up to 1.5-fold higher than those in wild type and all JM species remain detectable at late times (Figure 2B,C). Accumulation of SEIs may indicate a reduction in the efficiency with which the “second end” is engaged following formation of a stable interhomolog JM by the “first” or leading end. Alternatively, increased SEI levels in *dmc1 hed1* could be a consequence of higher than normal DSB formation (as discussed further below). Increased levels of mcJMs provide more specific support for defective coordination between DSB ends. This defect could result from enhanced strand-invasion activity of the “second” end when Dmc1 is absent, or a defect in the helicase-dependent mechanism that normally acts to resolve mcJMs [55]. Further evidence that coordination of DSB ends is defective in *dmc1 hed1* cells comes from the observation that non-allelic crossing-over between *HIS4::LEU2* and the native *LEU2* locus [56] is increased 3.2-fold (from 0.47% (±0.24 S.E.) in wild-type to 1.51% (±0.35 S.E.) in *dmc1 hed1*; non-allelic recombination bands are indicated by an asterisk in Figure 1B). In summary, analysis of JMs demonstrates that recombination is severely abnormal in *dmc1 hed1* cells. Most notably, strand-exchange now occurs primarily between sister-chromatids instead of homologs.

To demonstrate that results obtained at *HIS4::LEU2*, an artificial hotspot, were representative, we also examined dHJ intermediates and crossover products at *ERG1* (*YGR175c*), the site of a natural DSB hotspot on chromosome VII [57] (Figure 2D). In wild type cells, dHJ formation is biased to occur between homologs at the *ERG1* hotspot, with a IH/IS ratio of 4.5, similar to that seen at *HIS4::LEU2* (Figure 2E,F). This bias is again inverted in *dmc1 hed1* double mutants (IH-dHJ/IS-dHJ ratio of 0.15; Figure 2E,F). Crossover levels were also reduced at *ERG1* in *dmc1 hed1* cells, by 2.1-fold (Figure 2G,H). Thus, two recombination hotspots show dramatic reductions in homolog bias, but more modest reductions in final crossover levels in the *dmc1 hed1* double mutant.

Recombination and spore viability in *dmc1 hed1* was shown previously to be Rad51-dependent [47]. Further, Hed1 is known to inhibit Rad51-mediated strand-exchange via direct interaction [47], [48]. Using DNA physical assays at *HIS4::LEU2*, we confirmed that *rad51* mutation is fully epistatic to the *hed1* mutation indicating that Hed1 does not have a detectable influence on recombination in the absence of Rad51 (Figure S3).

### Recombination template choice is also altered when Rad51 strand-exchange activity is activated in the presence of Dmc1

Unexpectedly, the IH-dHJ/IS-dHJ ratio was also reduced in the *hed1* single mutant, in which Rad51 strand-exchange activity is activated in the presence of Dmc1. However, the effect was relatively mild compared to the *dmc1 hed1* double mutant (Figure 2B,C; Figure S1). In *hed1* cells, the IH-dHJ/IS-dHJ ratio was 2.1, down from 5.7 in wild type, but still much higher than the 0.22 ratio seen in *dmc1 hed1*. The less severe template choice defect of a *hed1* single mutant, compared to the *dmc1 hed1* double mutant, suggests that Dmc1 is, in effect, inhibitory to Rad51-mediated strand exchange (discussed further below).

### Dmc1 attenuates Rad51-mediated recombination

**Figure 3:**
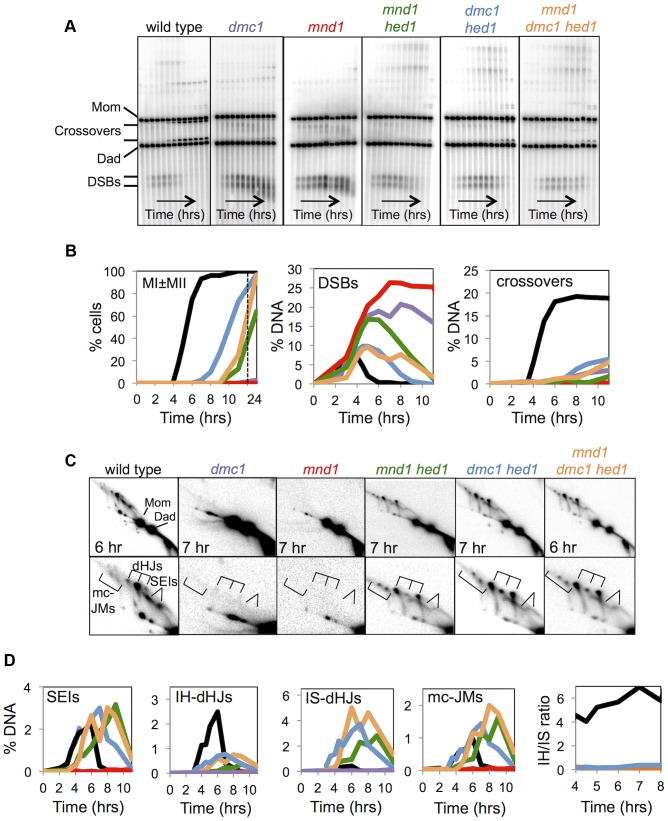
Mnd1 promotes Dmc1-dependent interhomolog recombination and Dmc1 inhibits Rad51-mediated recombination. A. Images of 1D Southern analysis at *HIS4::LEU2* for wild-type, *dmc1*, *mnd1*, *mnd1 hed1*, *dmc1 hed1*, and *mnd1 dmc1 hed1* time-course experiments. B. Quantification of the first meiotic division (MI±MII), DSBs, and COs for wild-type, *dmc1*, *mnd1*, *mnd1 hed1*, *dmc1 hed1*, and *mnd1 dmc1 hed1* time-course experiments. C. Images of representative panels from 2D Southern analysis with corresponding enlargements of the JM regions. D. Quantitation of JMs from 2D Southern analysis.

To further investigate the idea that Dmc1 is inhibitory to Rad51-mediated strand-exchange, we examined the effect of removing Hed1 when both Dmc1 and Rad51 have been incorporated into the recombination complex, but strand exchange is blocked. This phenotype is seen in the absence of the Hop2-Mnd1 complex [58], [59], [60], [61], [62], [63]. Similar to *dmc1* single mutants, *mnd1* mutants arrest during meiotic prophase with unrepaired DSBs (Figure 3A,B; Figure 1). However, the *mnd1* and *dmc1* phenotypes differ. First, arrest of *mnd1* mutants is more robust: 0% of *mnd1* cells have divided after 24 hrs compared to 3% of *dmc1* cells (Figure 3B; also see Figure 1C). Second, the extensive smearing of DSB signals seen in *dmc1* cells (indicative of extensive 5′-strand resection) is not observed in *mnd1* mutants (Figure 3A; also see Figure 1B). Third, crossover bands at *HIS4::LEU2* are not detected in *mnd1* cells while they reach 3% (∼15% of wild-type levels) in the *dmc1* mutant (Figures 3B; also see Figure 1C).

Like the *dmc1* mutant, arrest of *mnd1* cells is alleviated by *hed1* mutation, but much less efficiently (Figures 3B). By 11 hrs, 73% of *dmc1 hed1* cells have undergone one or both meiotic divisions, while only 13% of *mnd1 hed1* cells have divided. Crossover products in *mnd1 hed1* are also significantly reduced compared to *dmc1 hed1*, 1.7% versus 5.4%, respectively (Figure 3A,B).

Strikingly, analysis of JM intermediates in *mnd1 hed1* cells reveals an even stronger defect in template choice than that seen in *dmc1 hed1* (Figure 3C). JM formation is delayed by an additional 2 hrs in *mnd1 hed1* cells compared to *dmc1 hed1* cells and although IS-dHJs eventually form, IH-dHJs are barely detectable resulting in an IH-dHJ/IS-dHJ ratio of ≤0.12 (at least a 42-fold difference from wild type; Figure 3D). Thus, the presence of Dmc1 causes a general delay to Rad51-catalyzed JM formation and specifically blocks Rad51 filaments from catalyzing strand-exchange between homologs. Further support for this inference comes from analysis of an *mnd1 dmc1 hed1* triple mutant, which more closely resembles the *dmc1 hed1* double mutant than the *mnd1 hed1* double mutant with respect to JM timing as well as levels of IH-dHJs and crossovers (Figure 3C–D).

### Inhibition of Rad51 by Hed1 can facilitate interhomolog JM formation independently of Mek1

The meiotic recombination checkpoint pathway is important for normal template choice [32], [45], [56], [64], [65], [66], [67]. The phospho-kinase cascade that underlies this pathway leads to activation of the serine-threonine effector kinase, Mek1 [65], [67]. Absence of Mek1 kinase activity allows *dmc1* mutants to progress through meiosis and repair DSBs. However, both *mek1* and *dmc1 mek1* cells show severe defects in template choice, with recombination occurring primarily between sister chromatids [44], [66], [67].

**Figure 4:**
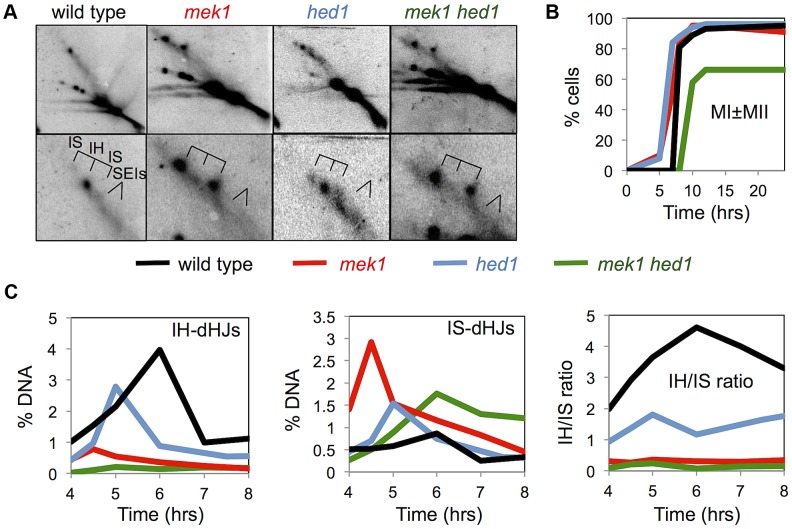
Mek1 and Hed1 make independent contributions to interhomolog template bias. A. Images of representative panels from 2D Southern analysis, with corresponding enlargements of the JM regions. B. Analysis of the first meiotic division in the indicated strains. C. Quantification of JM formation and IH/IS dHJ ratios in the indicated strains. Note that the signals trailing horizontally to the left of the parental species (“Mom” and “Dad”) results from overloading of the lanes. Samples were overloaded to aid detection of faint JM species. See Figure 1B for position of parental species.

To understand the relationship between Hed1-mediated repression of Rad51 and Mek1-mediated checkpoint signaling, we compared template choice in *mek1*, *hed1*, and *mek1 hed1* strains (Figure 4). Consistent with previous data [44], [66], JMs in *mek1* null mutants form primarily between sister chromatids resulting in an IH-dHJ/IS-dHJ ratio of 0.36 (Figure 4A–C). In the *mek1 hed1* double mutant, a further reduction in IH-dHJs is observed (Figure 4A), to the point where they are barely detectable above background, resulting in an IH-dHJ/IS-dHJ ratio of ≤0.12 (Figure 4C). Analysis of the ATP analog-sensitive *mek1-as* allele [68] gave results almost identical to those obtained with *mek1* null cells (Figure S4). These data indicate that, despite a severe template choice defect, *mek1* is not fully epistatic to *hed1*, i.e. Hed1 and Mek1 can function independently to promote interhomolog recombination.

### Chromosome pairing is defective in *dmc1 hed1* mutants

**Figure 5:**
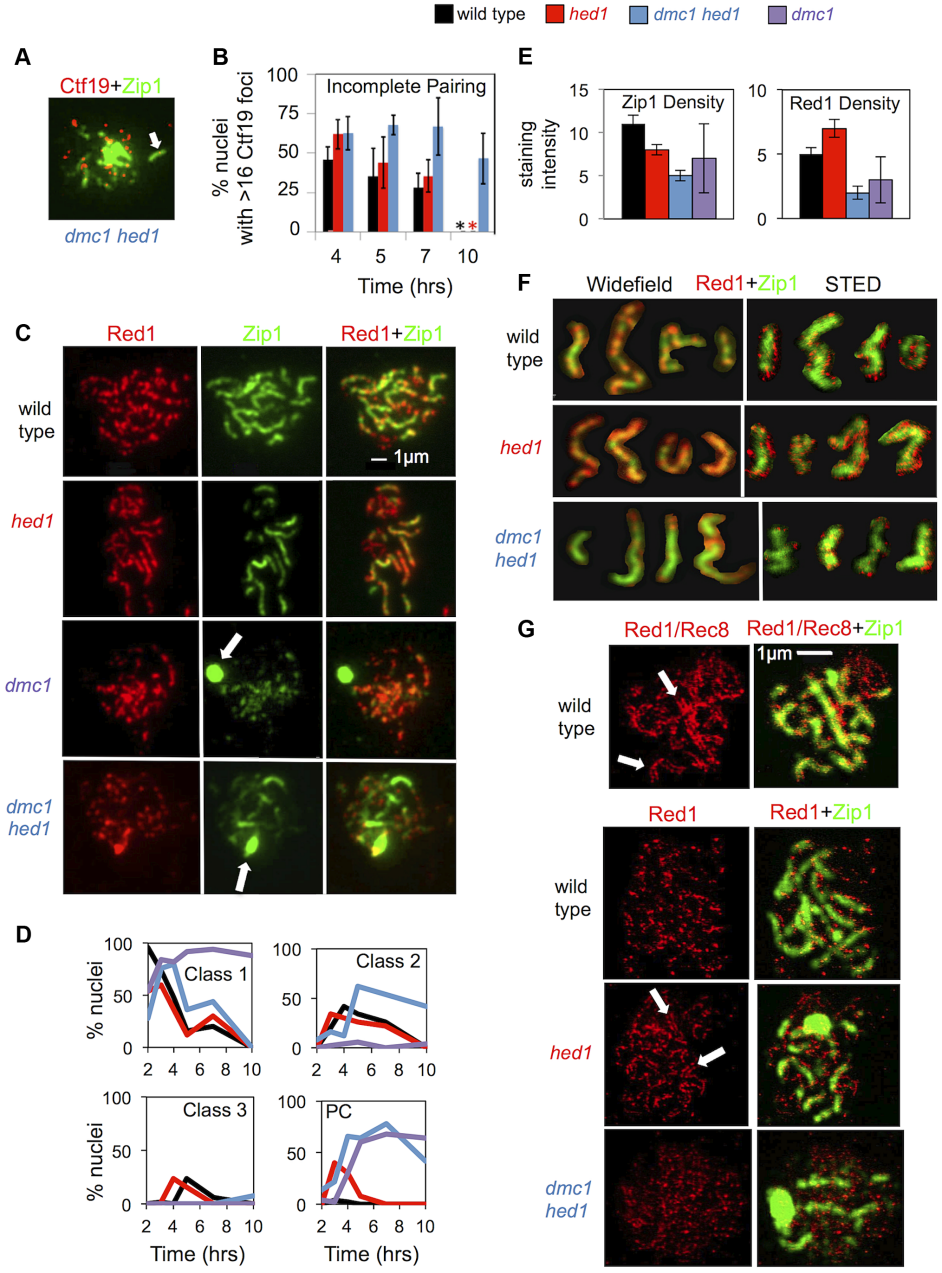
Synaptonemal complex formation is defective in *hed1* and *dmc1 hed1* strains. A. Representative image of a spread meiotic nucleus from the *dmc1 hed1* strain showing greater than 16 Ctf19 foci: immunostain for Zip1 is shown in green, Ctf19 stain is shown in red. Colocalization of a Ctf19 focus with an elongated Zip1 structure is indicated by an arrow. B. Quantitation of the percentage of prophase (Zip1 positive) nuclei displaying more than 16 Ctf19 foci as an indicator of the fraction of prophase nuclei containing one of more unpaired chromosomes, i.e. nuclei displaying “incomplete pairing.” Nuclei with fewer than 14 Ctf19 foci, which represented less than 15% of total Zip1^+^ nuclei, were excluded from the analysis. Approximately 50 nuclei were scored for each time point. The experiment was carried out 3 times; means and standard deviations are shown. Asterisks indicate that >95% of wild type and *hed1* single mutant cells had exited prophase by 10 hrs (most with evidence for insipient spore formation); these samples were not scored for Ctf19 foci. C. Representative images of spread nuclei immunostained for Zip1 in green and Red1 in red. Arrows indicate Zip1 polycomplexes (PC). The *dmc1* micrographs show a Class I nucleus (Zip1 puncta only) with a PC (white arrow); the *dmc1 hed1* nucleus is class II (Zip1 puncta and lines); and the wild-type and *hed1* nuclei are class III (predominantly linear Zip1 structures). D. Quantification of Zip1 staining classes over time. 50 nuclei were analyzed for each time point. E. Analysis of staining intensities of Red1and Zip1 in regions containing elongated Zip1 structures. Units are arbitrary. Quantitation can be found in Table S1. F. Representative images of individual spread chromosomes immunostained for Zip1 in green and Red1 in red from both wide-field and STED microscopy. Images are cropped and pasted on a black background. Note differences in the staining intensity of Red1 relative to Zip1. G. STED images of Red1 immunostaining (red). Corresponding Zip1 staining (green) was imaged at lower resolution via confocal microscopy. The top image pair is a wild-type nucleus stained with both Red1 and Rec8 primary antibodies and a secondary antibody that recognized both proteins shown in red. In the other 3 image pairs, the red signal is from anti-Red1 alone; no anti-Rec8 was used. Regions showing parallel linear rows of punctate staining for Rec8+Red1 (or Red1 alone) are indicated with arrows. Additional STED images are provided in Figure S5. Quantitation can be found in Table S2.

Given the importance of interhomolog recombination for chromosome pairing and synapsis, we monitored these processes in *hed1* and *dmc1 hed1* cells (Figure 5), which show, respectively, moderate and severe defects in interhomolog template choice (Figure 2). To monitor pairing, surface-spread meiotic nuclei were immunostained for the central kinetochore protein, Ctf19 (Figure 5A,B). When the 32 homologs of diploid budding yeast are completely paired, 16 Ctf19 foci are detected [69], whereas unpaired chromosomes result in nuclei with >16 foci. At 7 hrs, 28% of prophase wild-type nuclei display evidence of incomplete pairing compared to 63% of *dmc1 hed1* nuclei (Figure 5B). Furthermore, 46% of prophase *dmc1 hed1* nuclei have incompletely paired chromosomes even at 10 hours, a time when >95% of wild type and *hed1* single mutant cells have exited prophase (Figure 5B). These data indicate that chromosome pairing is inefficient in *dmc1 hed1* cells. In contrast, Ctf19 immunostaining of *hed1* single mutant cells indicates little or no defect in the efficiency of pairing (Figure 5B).

### Defective morphogenesis of synaptonemal complexes in *hed1* mutants

SC morphogenesis was monitored by immunostaining spread nuclei for Red1, a component of the Mek1 signaling pathway that localizes along SC axial/lateral elements, and for Zip1, a major component of the SC central region (Figure 5C) [70], [71]. Nuclei were assigned to one of three structural classes [70] (Figure 5D). Class 1 nuclei, corresponding to the stage called leptonema, contain only Zip1 puncta, but no elongated Zip1 structures; Class 2 nuclei (zygonema, partial synapsis) have at least one elongated Zip1 structure together with multiple Zip1 puncta; and Class 3 (pachynema, full synapsis) defines nuclei in which the majority of Zip1 staining forms elongated structures. Nuclei were also analyzed for the presence of extra-chromosomal aggregates of Zip1 called polycomplexes, a typical manifestation of defective SC assembly (highlighted by arrows in Figure 5C) [12], [72].

Although the kinetics of SC formation were similar for both wild type and *hed1* strains, the *hed1* mutant showed unexpected defects in SC morphogenesis. Polycomplexes, while rarely seen in wild type (≤4% of cells at any single time point), were present in up to 40% of *hed1* nuclei (Figure 5D). Furthermore, the average intensity of Zip1 staining along SCs was 1.4 fold lower than that of wild type, while Red1 staining intensity along SCs was 1.4 fold higher (Figure 5E; Table S1). In addition, the pattern of Red1 and Zip1 abundance along SCs was altered (Figure 5C,F). Although SCs appear rather uniform when viewed by metal staining and electron microscopy, immunostaining for individual components reveals alternating domains of high and low abundance [73], [74]. This domain substructure is observed for a number of chromosomal proteins including Zip1, Red1 and its associated axial protein Hop1, and the meiosis-specific cohesion component, Rec8. Domains of abundant Zip1 and Rec8 alternate with domains of abundant Hop1 and Red1 [73], [74].

Consistent with published data, in wild-type nuclei, Red1 and Zip1 form alternating domains of relative abundance ([73]; Figure 5F). In *hed1* nuclei, the Red1-Zip1 domain structure was less pronounced. This appears to be a consequence of the overloading of Red1 at regions where it would normally be found in low abundance (Figure 5C–F). These data link inhibition of Rad51-mediated inter-sister recombination to normal axis morphogenesis. This domain “blurring” is reminiscent of the SC defects seen in cells lacking the AAA+ ATPase, Pch2 [73], [74].

To examine Red1 localization in more detail, we employed stimulated emission depletion microscopy (STED), a method of light microscopy that can achieve 50 nm resolution compared to the 200 nm resolution provided by standard widefield epifluorescence microscopy [75]. As a proof-of-principle, we first used a mixture of antibodies against Red1 and Rec8 to achieve a uniformly dense staining pattern along SC axial/lateral elements. This approach revealed parallel rows of densely-spaced foci separated by ∼100 nm (Figure 5G, white arrows). Thus, Red1 and Rec8 remain closely associated with SC lateral elements during the spreading procedure and parallel lateral elements can be resolved by STED microscopy.

STED imaging of nuclei stained for Red1 alone resolved the 30–40 abundance domains of Red1 per nucleus, into 217±32 staining foci ranging from simple puncta to elongated structures with lengths of up to 600 nm. Thus, abundance domains observed in spread preparations are composed of clusters of about 5–7 discrete Red1-containing complexes rather than a single axis-associated structure or a more uniformly dispersed distribution of protein molecules along axial/lateral elements (Figure 5F,G; Figure S5; Table S2). Consistent with results of conventional microscopy, the density of Red1 staining foci along SCs detected via STED was higher in *hed1* single mutants than in wild type. Indeed, the increased density of Red1 puncta resulted in regions where the parallel configuration of the underlying lateral elements was evident (as observed in the Red1+Rec8 mixed staining experiment) again demonstrating that Red1 is a component of axial/lateral elements (Figure 5F,G; Table S2).

### Synapsis is inefficient and abnormal in *dmc1 hed1* cells

To address whether activation of Rad51 restores synapsis to *dmc1* mutants, we monitored SC formation in *dmc1* and *dmc1 hed1* cells (Figure 5C,D). Primarily Class 1 nuclei are observed in *dmc1* nuclei, 68% of which contain a large Zip1 polycomplex. This severe SC defect is partially suppressed by *hed1* mutation, with 62% of *dmc1 hed1* nuclei progressing to Class 2, and 8% to Class 3 (Figure 5C,D). However, despite the large fraction of Class 2 nuclei, the average number of elongated Zip1 structures in this class was 2.4 fold lower in *dmc1 hed1* than in wild-type nuclei (Table S1). Furthermore, *dmc1 hed1* nuclei show a unique morphological abnormality, with decreased Red1 staining intensity along SCs and a dramatic “blurring” of the alternating high and low abundance domains of Red1 and Zip1 (Figure 5E,F; Table S1). Analysis of STED images confirmed that the density of Red1 puncta is lower in the *dmc1 hed1* double mutant than in wild type (Figure 5F,G; Figure S5; Table S2). Thus, Dmc1 influences the composition of axial/lateral elements.

Because Zip1 can form elongated structures that are not associated with chromosomes [72], it was important to confirm that the Zip1 structures formed in *dmc1 hed1* nuclei are *bona fide* SCs. To this end we showed that the Zip1 structures colocalize with both centromeres (by co-staining with the centromere-associated protein, Ctf19) and the crossover-associated protein, Zip3 (Figure 5A; Figure S6). More definitively, electron microscopy of silver-stained chromosome spreads from *dmc1 hed1* revealed the typical SC tripartite structure (Figure S6). Thus, SCs visualized by metal staining and electron microscopy can appear quite normal despite having an abnormal composition with respect to the abundance and arrangement of Red1 and Zip1. We conclude that activation of Rad51's strand invasion activity can promote assembly of SCs in the absence of Dmc1. However, the composition of axial/lateral elements is abnormal and SC morphogenesis is delayed and, typically, incomplete.

### Chromosome-wide genetic map distances in *hed1* and *dmc1 hed1* mutants are similar to wild type

**Figure 6:**
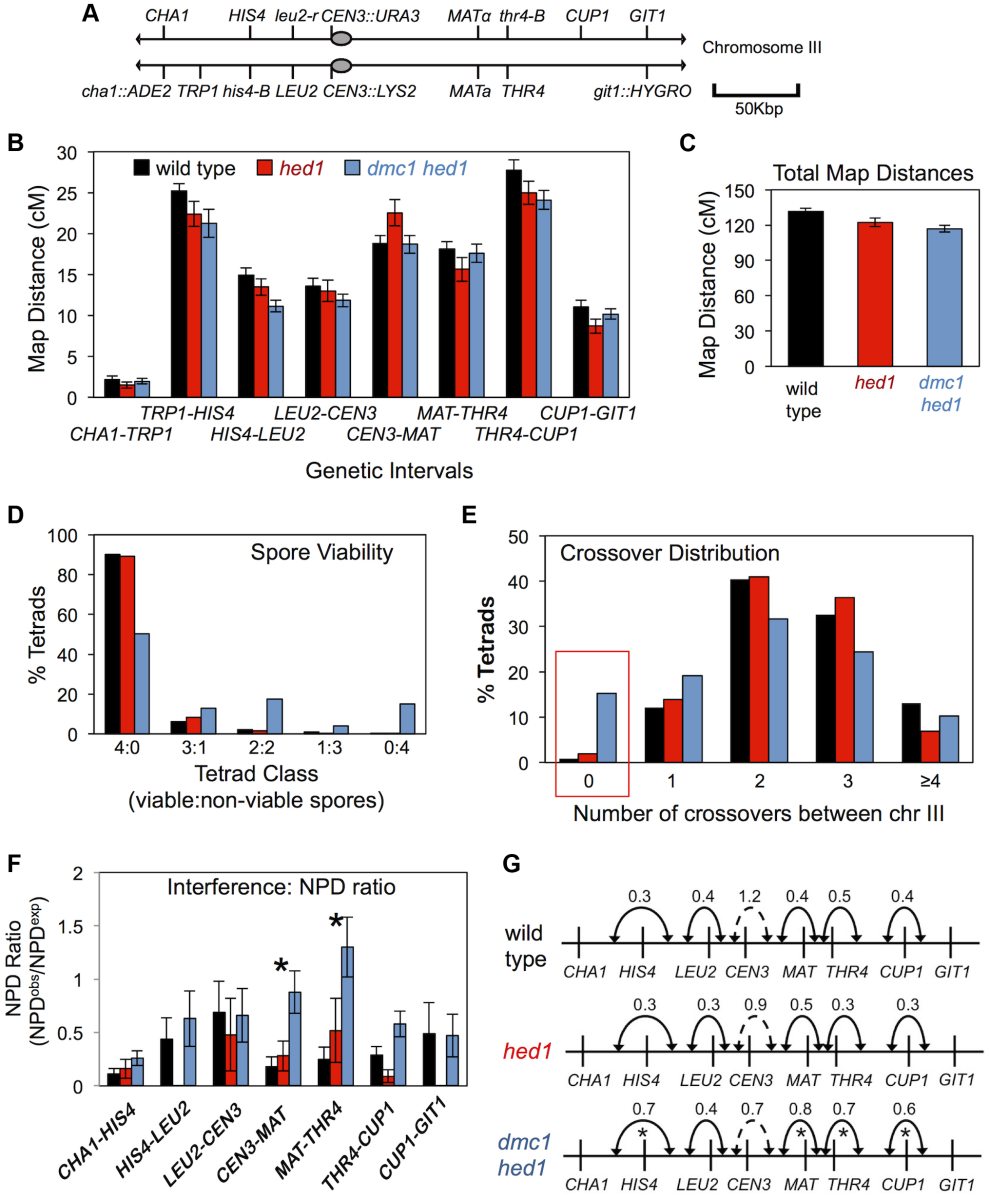
Analysis of crossing-over in *hed1* and *dmc1 hed1* mutants. A. Map of marker configurations along chromosome III. Note that this strain does not carry the artificial *HIS4::LEU2* hotspot. B. Map distances for 8 genetic intervals along chromosome III. Map distances were calculated via the method of Perkins (T+3NPD/total ×100). Sample sizes were 1039, 487, and 1538 tetrads with four viable spores for wild type, *hed1*, and *dmc1 hed1* respectively. Error bars represent standard error. C. Cumulative chromosome III map distances. D. Distribution of tetrads with various numbers of viable spores. Spore viabilities are 96% (4535/4716) in wild type, 96% in (2106/2184) *hed1*, and 70% (8523/12208) in *dmc1 hed1*. E. Distribution of chromosome III crossover classes. The red box highlights E0 tetrads that lack crossovers between chromosome III. F. NPD ratios for 6 intervals along chromosome III. For this analysis, the *CHA1-TRP1* and *TRP1-HIS4* intervals were combined. Error bars represents standard error. See Table S3 for NPD ratios and statistical tests. Asterisks mark intervals in which the reduction in the strength of interference relative to that in wild-type cells is significant. G. Map distance ratios (Adj^CO^/Adj^PD^) for adjacent intervals. Numbers shown are averages of the two Adj^CO^/Adj^PD^ ratios derived for reciprocal analysis of each adjacent interval pair. Solid lines indicate adjacent intervals for which significant positive interference was detected; dashed lines indicate that the ratio is not significantly different from 1, i.e. no interference. Asterisks mark interval pairs for which the reduction in the strength of interference relative to that in wild-type cells is significant. See Table S4 for map distances and statistical testing.

Although crossing-over at the *HIS4::LEU2* and *ERG1* hotspots is reduced, respectively, by 3.5 and 2.1-fold, (Figures 1 and 2) in *dmc1 hed1* cells, spore viability remains relatively high (∼70%; see below; [47]). A possible explanation is that despite a >2-fold reduction in crossing-over, crossover assurance remains efficient in *dmc1 hed1* cells. To address this possibility we used tetrad analysis to determine crossover frequencies and distributions for eight linked intervals spanning the entire length of chromosome III (Figure 6A). Surprisingly, and despite the defects in template choice, homolog pairing and synapsis, genetic map distances for the *hed1* single mutant and the *dmc1 hed1* double mutant were very similar to those of wild type (Figure 6B).

In the *hed1* mutant, minor fluctuations in map distances were detected in some intervals, but the overall map distance for chromosome III was nearly identical to that of wild type (Figure 6B,C). In the *dmc1 hed1* double mutant, small but significant decreases (13–25%) in map distances were detected in three of eight intervals, but overall map distance of chromosome III was reduced by only 11%, from 131.6 cM (2.6 crossovers per meiosis) in wild type to 116.8 cM (2.3 crossovers per meiosis) in *dmc1 hed1* (Figure 6B,C). Thus, with respect to crossover frequency measured by tetrad analysis, Rad51 is ostensibly capable of efficiently substituting for Dmc1.

### Crossover control is defective in the *dmc1 hed1* strain

Spore viability of *dmc1 hed1* cells is only 70% compared to 96% for both wild type and the *hed1* single mutant [47]. The spore viability pattern of the *dmc1 hed1* strain is indicative of meiosis I nondisjunction, with a preponderance of tetrads containing two and zero viable spores (Figure 6D). Allelic, centromere-linked *URA3* and *LYS2* markers on chromosome III were used to confirm an elevated rate of meiosis I nondisjunction. Disomic spores carrying both *CEN3::URA3* and *CEN3::LYS2* were observed in only 0.4% of wild-type tetrads (5/1179) compared to 1.5% of *dmc1 hed1* tetrads (45/3052).

Reasoning that the reduced spore viability of *dmc1 hed1* mutants may be caused by defective crossover assurance, we determined the frequency of tetrads in which chromosome III failed to undergo any crossing-over (Figure 6E). If crossovers between chromosome III were randomly distributed in wild-type cells, 8% of tetrads would have zero crossovers between chromosome III. On the contrary and as expected, crossover assurance is efficient in wild type, with only 0.9% (9/1039) of tetrads lacking a crossover between chromosome III (for a random distribution, the probability of ≤9 non-exchange tetrads occurring given a sample size of 1039 is <0.0001).

Remarkably, even though the average crossover frequency in *dmc1 hed1* cells is very similar to wild type (2.3 versus 2.6 crossovers per chromosome III per meiosis), 15% (210/1376) of tetrads lack a crossover between chromosome III (Figure 6E). Not only is this level of crossover failure much higher than that of wild-type cells (P<0.0001), but it is also significantly higher than that predicted for a Poisson distribution (10% of tetrads; P<0.0001). Thus, despite a high level of crossing-over in the population, crossover assurance is profoundly defective in *dmc1 hed1* cells. The frequency of non-exchange tetrads seen in *hed1* single mutants (2%) may by slightly higher than that of wild type (0.9%), but the difference is not significant in this data set.

Consistent with the possibility that crossover failures in *dmc1 hed1* cells results from defective homolog pairing (Figure 5), gene conversion events were almost undetectable in non-exchange tetrads. Only 0.5% of tetrads that lack an exchange between chromosome III (1/210) had a detectable gene conversion for markers along that chromosome. By comparison, 8% of all *dmc1 hed1* tetrads had a detectable gene conversion between chromosome III (94/1185; P<0.0001).

A second aspect of crossover control, interference, was analyzed by a new approach that took advantage of the ability to purge the data of tetrads in which chromosome III did not undergo a crossover event. This approach avoids the diminution of the genetic signature of interference that is associated with defective crossover assurance [76] (see Supplemental Materials, Figure S7, for a comparison of this approach with that of conventional analysis of interference; see Table S5, Figure S8 and Text S1 for further validation of the approach).

Interference was first assessed by analyzing frequencies of non-parental di-type (NPD) tetrads. NPDs arise from a double crossover event within a single genetic interval that involves all four chromatids. The expected number of NPD tetrads is calculated assuming the absence of crossover interference [77]. Ratios of observed/expected NPDs that are significantly <1 indicate positive crossover interference. In wild-type tetrads, NPD ratios were <1 for all seven intervals tested and significant positive crossover interference was inferred in four intervals (Figure 6F; Table S3). In contrast, NPD ratios in *dmc1 hed1* tetrads showed a general increase relative to wild type and significant interference was detected in only one of the seven intervals indicating substantially weaker crossover interference (Figure 6F; Table S3). Mutation of *hed1* alone did not cause a detectable change in crossover interference relative to wild type.

Weakened interference in *dmc1 hed1* cells was confirmed by analysis of coincident crossovers in adjacent genetic intervals [78]. For a given reference interval, tetrads are first divided into two subsets: those with a crossover (AdjCO) in the interval and those without (AdjPD). These two subsets are then analyzed for map distances in an adjacent interval (test interval) and interference is expressed as the ratio of these two distances, which is indicative of the strength of interference (cM^AdjCO^/cM^AdjPD^ in Figure 6G). By this method, significant interference (cM^AdjCO^/cM^AdjPD^<1) was detected for 5 of 6 intervals in wild type, *hed1*, and *dmc1 hed1* indicating that significant interference can occur in the absence of both *hed1* and *dmc1*. However, *dmc1 hed1* shows significant decreases in the strength of interference in 4 of the 6 intervals tested (Figure 6G; Table S4). We conclude that crossover interference is reduced, but not eliminated in *dmc1 hed1* tetrads.

### Elevated crossover bias partially compensates for the template choice defect of *dmc1 hed1* mutants

**Figure 7:**
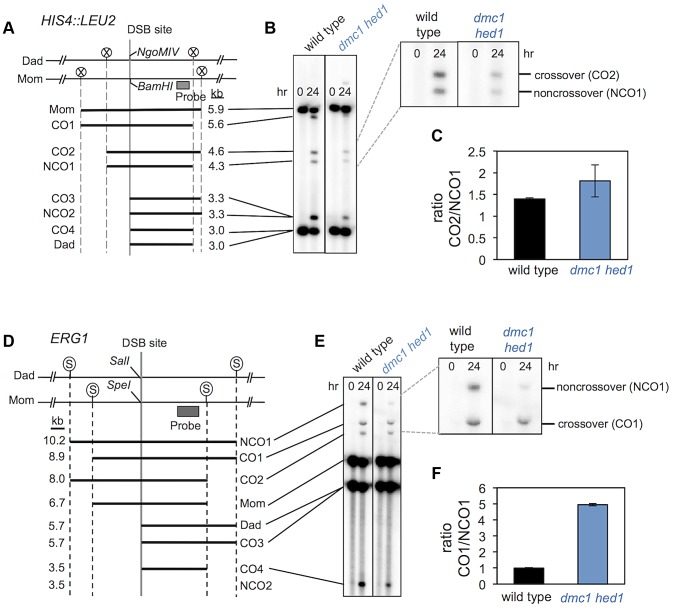
*dmc1 hed1* strains have elevated crossover/non-crossover ratios at two recombination hotspots. A. Map of the *HIS4::LEU2* DSB site showing diagnostic restriction site heterologies. Positions of *Xho*I (circled Xs), *Bam*HI and *Ngo*MIV sites are indicated. B. 1D Southern analysis of *Xho*I+*Ngo*MIV doubly digested genomic DNA isolated at 0 and 24 hrs after induction of meiosis. The magnified image highlights bands that are specifically diagnostic of crossovers and noncrossovers. C. Crossover/non-crossover ratios at *HIS4::LEU2* calculated from quantification of four independent sets of time courses. Error bars indicate standard error. D. Map of the *ERG1* DSB site showing diagnostic restriction site heterologies. Positions of *Sac*II (circled Ss), *Sal*I and *Spe*I sites are indicated. E. Southern analysis of *Sac*II+*Sal*I doubly digested genomic DNA isolated at 0 and 24 hrs after induction of meiosis. The magnified image highlights bands that are specifically diagnostic of crossovers and noncrossovers. F. Crossover/non-crossover ratios at *ERG1* calculated from quantification of four independent sets of time courses. Error bars indicate standard error.

Paradoxically, even though homolog bias and chromosome pairing/synapsis are severely defective in *dmc1 hed1* cells, the overall crossover rate is similar to that of wild-type cells (Figure 6). A possible explanation is that a larger fraction of interhomolog recombination events develop into crossovers rather than non-crossovers in *dmc1 hed1* cells. To test this possibility, we measured the ratios of interhomolog crossovers and non-crossovers at the *HIS4::LEU2* and *ERG1* loci (Figure 7). Restriction site polymorphisms at these two sites allow crossover and non-crossover products to be distinguished in Southern blot assays [41].

At *HIS4::LEU2*, the ratio of crossovers to non-crossovers in wild-type cells is 1.4, while in *dmc1 hed1* cells the crossover/non-crossover ratio is increased to 1.8 (Figure 7C). More dramatic results were obtained at the *ERG1* hotspot, where the crossover/non-crossover ratio is 1.0 in wild-type cells and 4.9 in *dmc1 hed1* cells (Figure 7D–F). These data suggest that the crossover versus non-crossover outcome of meiotic recombination can be dynamically regulated in response to fluctuations in the number of interhomolog interactions. However, given the severity of the homolog template bias defect, the observed increases in the crossover/non-crossover ratios do not fully account for the relatively minor crossover defect seen in *dmc1 hed1* cells (see Supplementary Text for relevant calculations). This suggests that enhancement of the crossover versus non-crossover outcome is not the only mechanism that compensates for the homolog bias defect of *dmc1 hed1* (see Discussion).

### Crossover bias is maximized in *dmc1 hed1* cells

Martini et al. previously demonstrated that global reductions in DSB levels, caused by hypomorphic alleles of *SPO11*, are compensated for by increases in crossover/non-crossover ratios [18]. This study led to the concept that crossover frequency is under homeostatic control such that cell-to-cell variance is low despite relatively high variance in DSB numbers [15], [17], [21]. We reasoned that homeostatic control responds to the number of interhomolog recombination interactions and not the number of DSBs per se. Thus, we predicted that *dmc1 hed1* cells would be unable to compensate for even mild reductions in DSB levels.

To test this prediction, we determined the effects of combining *dmc1 hed1* with hypomorphic *spo11* mutations. Crossover/non-crossover ratios were determined using a previously described random spore assay that measures the crossover frequency associated with intragenic recombination at the *ARG4* locus [18].

**Figure 8:**
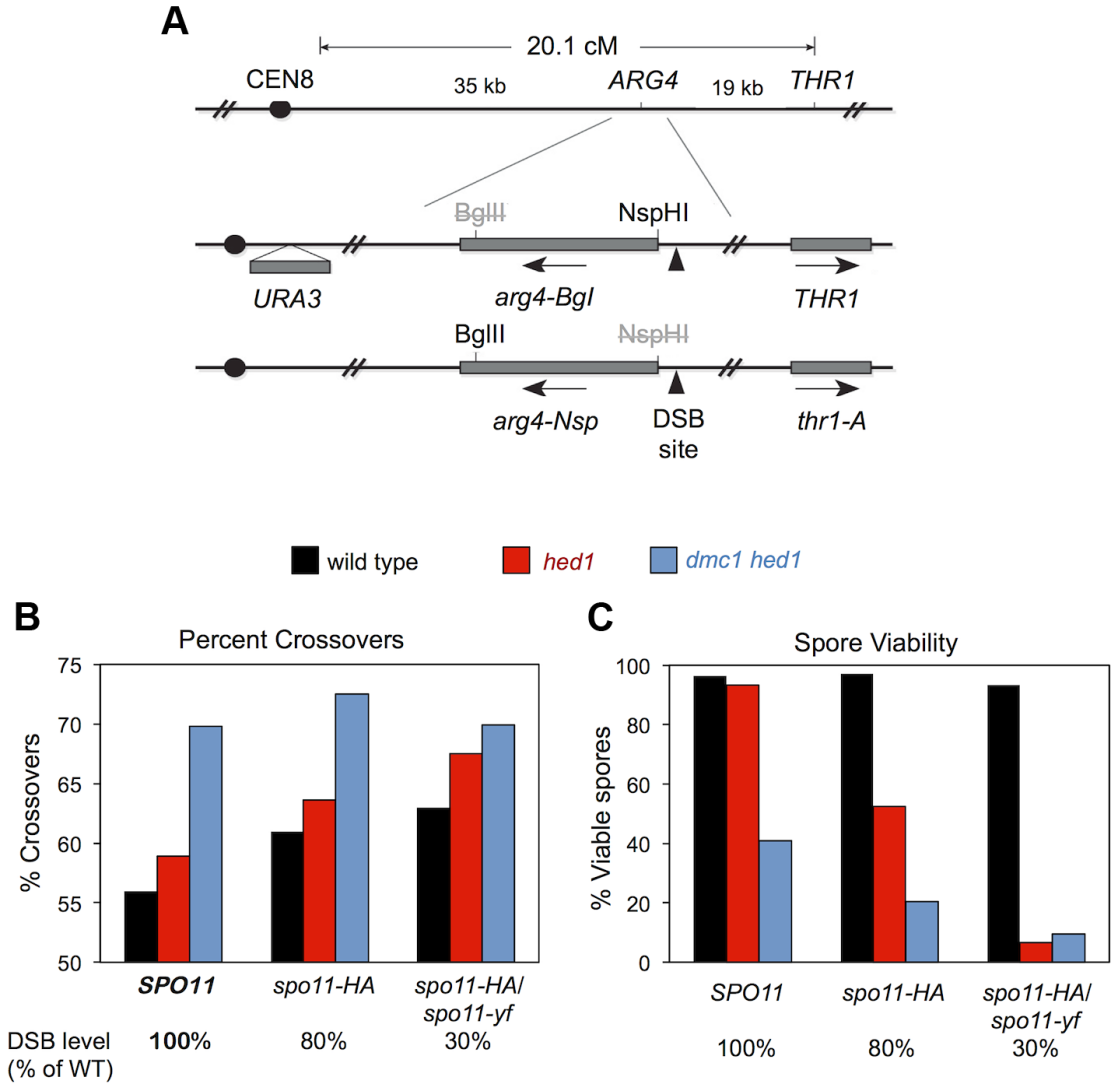
Crossover/noncrossover ratios at *ARG4* measured by random spore analysis. A. Map of the *ARG4* region (top) and heterozygous markers used to select *ARG4^+^* gene conversions and determine whether they are associated with crossing over (bottom). B. Percent of *ARG4* gene conversions associated with crossing over. C. Percentage of viable spores for the strains analyzed in (B).

We constructed sets of *dmc1 hed1* mutant strains and *DMC1^+^ HED1^+^* control strains that were also homozygous for *SPO11^+^* (wild type), or *spo11-HA*, or heterozygous for *spo11-HA*/*spo11-yf*; these combinations of *SPO11* alleles result in 100%, ∼80%, and ∼30% of normal DSB levels, respectively [18]. In the *DMC1^+^ HED1^+^* control strains, the fraction of recombination events that result in crossing-over increases with decreasing DSB levels [18]; the crossover/non-crossover ratios in *SPO11^+^, spo11-HA*, and *spo11-HA*/*spo11-yf* strains are 1.3 (56% crossovers), 1.6 (61%), and 1.7 (63%), respectively (Figure 8). Consistent with our analysis at the *HIS4::LEU2* and *ERG1* hotspots (Figure 7), the crossover/non-crossover ratio in the *dmc1 hed1 SPO11^+^* strain is already very high (2.3; 70% crossovers). This ratio may increase slightly in the *spo11-HA* background (2.6; 72.5% crossovers), but no increase was seen for the most severe *spo11* allele combination, *spo11-HA*/*spo11-yf* (2.3; 70% crossovers).

Thus, homeostatic mechanisms that modulate the crossover/non-crossover ratio appear to be already operating at maximum capacity in *dmc1 hed1* cells and therefore are unable to further compensate for reduced DSB levels. Consistent with this interpretation, in the *dmc1 hed1* background, *spo11-HA* caused a 2-fold drop in spore viability (from 41% to 20% viable spores; n = 140 and 180 tetrads, respectively) and the *spo11-HA*/*spo11-yf* combination resulted in a 4.3-fold decrease (9.5% viable spores; n = 100 tetrads; Figure 8C). In sharp contrast, the spore viabilities of *DMC1^+^ HED1^+^* control strains were not significantly impacted by the *spo11-HA* and *spo11-HA*/*spo11-yf* mutations (spore viabilities of 97%, 97%, and 93% for *SPO11, spo11-HA,* and *spo11-HA*/*spo11-yf* strains, respectively; n = 100 tetrads for all three strains; Figure 8C).

In the *hed1* single mutant background, crossover/non-crossover ratios are slightly higher than those of wild type and increase with decreasing DSB levels. The crossover/non-crossover ratio was 1.4 (59% crossovers) in *hed1 SPO11* and increased to 1.8 (64% crossovers) in *hed1 spo11-HA* and 2.1 (68% crossovers) in *hed1 spo11-HA/spo11-yf* (Figure 8B). Surprisingly, spore viability decreased from 93% in *hed1 SPO11^+^* to 53% in *hed1 spo11-HA* and only 6.5% in *hed1 spo11-HA/spo11-yf* (Figure 8C). These data indicate that Hed1 helps cells compensate for suboptimal DSB levels and imply that wild-type levels of interhomolog bias are important for efficient crossover homeostasis.

### The differential impact on local versus global crossing-over implies a second homeostatic process is operating in *dmc1 hed1* cells

**Figure 9:**
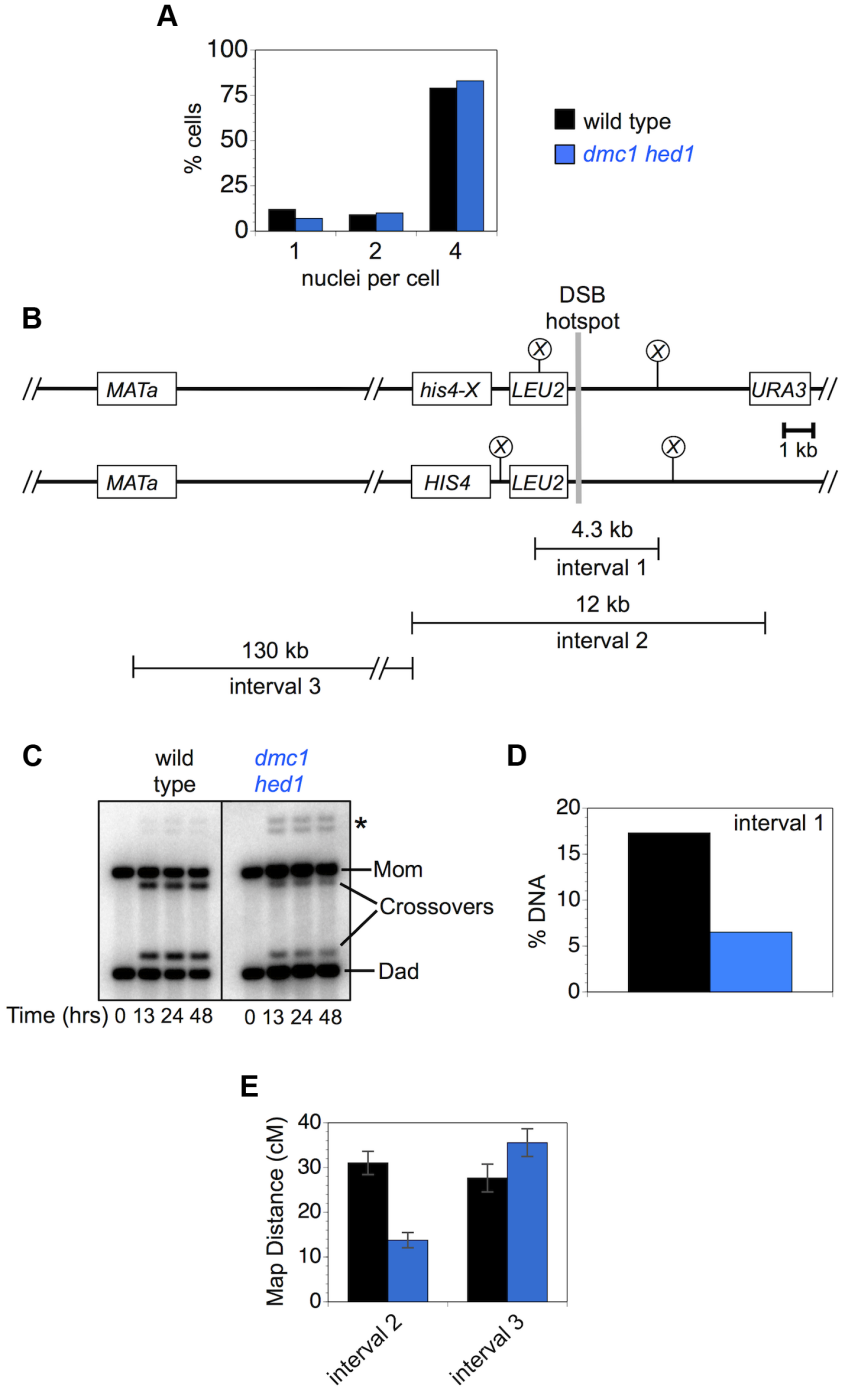
Crossover homeostasis as a function of interval size. A. Efficiency of meiotic divisions in parallel cultures of wild-type and *dmc1 hed1* cells. B. Physical map of *HIS4::LEU2* and adjacent regions of chromosome III showing intervals monitored for crossing-over. Crossovers in interval 1 were monitored by Southern analysis (as in Figure 1A). Crossovers in intervals 2 and 3 were monitored by tetrad analysis. C. 1D Southern analysis of crossing-over in parallel cultures (analyzed in A) of wild-type and *dmc1 hed1* cells. Asterisk highlights bands diagnostic of non-allelic recombination between *HIS4::LEU2* and *leu2::hisG* located ∼25 kb away at the native *LEU2* locus. D. Quantification of the Southern image shown in (C). Crossover levels at 48 hrs are shown. E. Map distances for intervals 2 and 3 calculated from tetrads sampled from the same cultures analyzed in A, C and D.

Although chromosome-wide crossover levels in *dmc1 hed1* cells are similar to wild type (Figure 6), crossing-over at both the *HIS4::LEU2* and *ERG1* hotspots is substantially reduced [47] (Figures 1, 2). A trivial explanation for this disparity is that dissected tetrads are enriched for crossovers relative to the cell population as a whole. This possibility was addressed by measuring crossover levels by both Southern blot and tetrad analysis in cells sampled from the same meiotic cultures (Figure 9). The two selected cultures underwent meiotic divisions with nearly identical efficiencies and formed similar fractions of mature asci containing four spores (Figure 9A and data not shown). Similar to the data in Figure 1, Southern analysis of crossovers within the 4.3 kb *Xho*I fragment surrounding the *HIS4::LEU2* DSB site (“interval 1”) were reduced 2.7-fold from 17.3% in wild-type cells to 6.5% in *dmc1 hed1* cells (Figure 9A,B,C). By tetrad analysis, crossing-over within the larger 12 kb *URA3* to *HIS4::LEU2* interval surrounding the *HIS4::LEU2* DSB site (“interval 2”) was reduced 2.2-fold, from 31.0 cM to 13.8 cM (Figure 9D). At 130 kb, the adjacent interval, from *HIS4::LEU2* to *MAT* (“interval 3”), is physically just over ten times larger than interval 2, but shows a very similar crossover rate (27.7 cM in wild type). Strikingly, crossing-over in interval 3 is not reduced in *dmc1 hed1* cells (Figure 9E). In fact, the map distance is significantly increased relative to wild type (35.6 cM in *dmc1 hed1*).

Thus, in *dmc1 hed1* cells, interhomolog interactions at DSB hotspots appear to be diminished to the point where homeostatic mechanisms are unable to maintain local crossover levels. However, crossover levels measured over larger distances remain similar to wild type. These data suggest that a second homeostatic mechanism operates over large chromosomal regions to help maintain crossover levels in *dmc1 hed1* cells (discussed below).

## Discussion

### Activated Rad51 does not effectively substitute for Dmc1

Previous work showed that activation of Rad51's strand-exchange alleviates the meiotic arrest of *dmc1* cells and results in near normal levels of crossing-over and high spore viability [45], [47]. A possible interpretation of these data is that Rad51 normally contributes the bulk of strand exchange activity to the meiotic recombination process while Dmc1 acts as a regulatory factor [47]. On the contrary, more recent work indicates that Rad51's strand exchange activity is dispensable for meiotic recombination [46], but Rad51 plays an essential supporting role for Dmc1-mediated strand-exchange [1], [32], [46], [79], [80]. Consistent with these conclusions, the current work shows that Rad51-mediated meiotic recombination is profoundly abnormal, but near normal levels of crossing-over still form by virtue of compensatory mechanisms (discussed below). Rather than catalyzing homology search and strand invasion, current and previous analyses indicate that Rad51 complexes normally play multiple regulatory roles during meiotic prophase, including: (i) facilitating the assembly of Dmc1 nucleoprotein filaments [4], [46], [81]; (ii) limiting the extent of DSB resection [32], [81]; (iii) enhancing Dmc1-dependent interhomolog template bias [32], [46]; and (iv) promoting normal SC morphogenesis.

**Figure 10:**
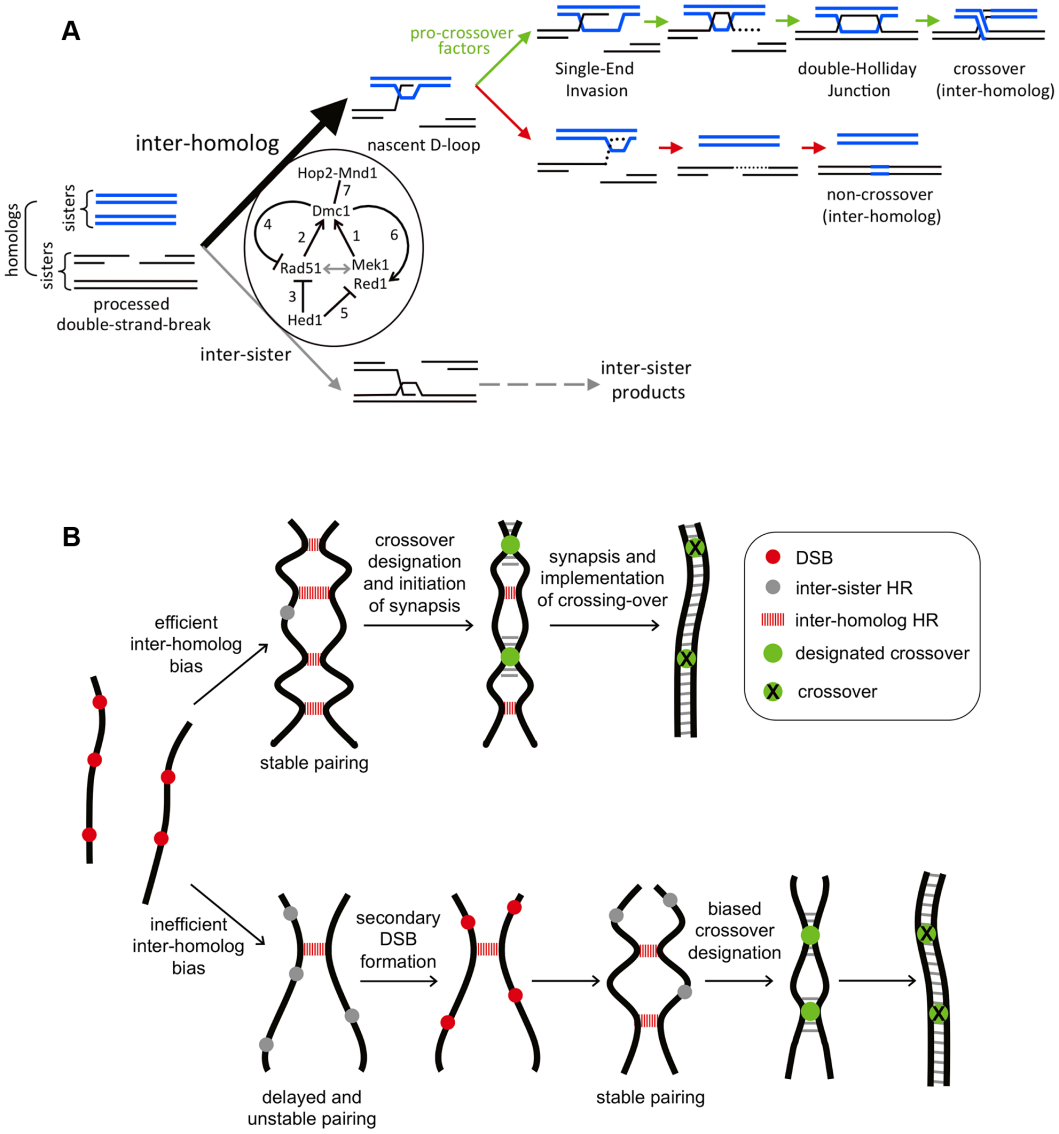
Models for Rad51-Dmc1 mediated interhomolog bias and homeostatic responses to inefficient interhomolog interactions. A. Rad51-Dmc1 dependent interhomolog recombination during meiosis. DNA intermediates leading to interhomolog or intersister recombination are shown. Intersister strand-exchange is presumed to produce intermediates similar to those shown for interhomolog recombination. The 5-fold interhomolog template bias detected in wild-type cells is achieved by the illustrated interactions, as follows: (1) Mek1-Red1 (and presumably Hop1) function together with Rad51 filaments (2) to facilitate filament assembly and interhomolog strand-exchange activity of Dmc1. The grey double arrow indicates interdependence between these two ensembles. (3) Hed1 inhibits Rad51's strand exchange activity and may also influence the stability of Rad51 filaments [44]. However, *in vivo*, Hed1 is not the major inhibitor of Rad51-mediated strand-exchange, which is largely inhibited by Dmc1 (4). Moreover, Hed1 is not required for the Dmc1-accessory role of Rad51 (2). Hed1 also limits the loading of Red1 onto homolog axes (5). Conversely, Dmc1 promotes Red1 loading (6) suggesting a positive feedback loop between Mek1-Red1 and Dmc1. Finally, Hop2-Mnd1 is essential for Dmc1-mediated interhomolog strand-exchange (7). B. Homeostatic responses compensate for inefficient interhomolog bias. Homologs are represented by thick black lines. Top row: efficient interhomolog bias results in stable pairing and SC formation ensues. Coincident designation of crossover sites and associated interference results in the normal number and distribution of crossovers. If interhomolog bias is inefficient, as seen in *dmc1 hed1* cells, pairing and synapsis are delayed and inefficient. Persistent checkpoint signaling resulting from delays in both synapsis and DSB-repair provoke secondary DSB formation and additional interhomolog interactions until stable homolog pairing and synapsis are achieved. Stochastic failure of this process is responsible for crossover failures (non-exchange tetrads). Although the final number of interhomolog interactions is lower than normal, biased outcome results in normal crossover frequencies.

The precise spatial arrangement of Rad51 and Dmc1 on DSB-ends remains unclear. Cytological experiments argue against mixed filaments of Rad51 and Dmc1 [80], [82]. Instead, immunostaining patterns suggest that homo-filaments of the two strand exchange proteins are assembled as immediately adjacent pairs. One interpretation of side-by-side foci is that Dmc1 assembles on one DSB-end and Rad51 on the other [78]. This idea has proven attractive and is suggested to account for a variety of observations [1], [44], [54], [82], [83]. Under this scenario, while the Rad51-associated end is rendered inactive for strand invasion, it still functions in a regulatory capacity to block access to the sister-chromatid template by the Dmc1-associated end and/or acts to antagonize the inhibitory effects of cohesin on interhomolog recombination [44]. However, the demonstration that Rad51 enhances the assembly and strand exchange activity of Dmc1 makes it likely that Dmc1 and Rad51 can assemble on the same DNA end [46], [79]. Thus, paired Rad51-Dmc1 foci may represent a single DSB-end. This configuration can accommodate the unexpected observation that Dmc1 complexes are generally inhibitory to Rad51-mediated strand exchange (Figure 3; see model in Figure 10A). We suggest that assembly of Dmc1 at a DSB-end modulates the adjacent Rad51 filament in such as way that its strand-exchange activity is inhibited. Defining the precise arrangement of Rad51 and Dmc1 on DSB ends *in vivo* remains a key objective for understanding meiotic recombination.

Consonant with the fact that Hop2-Mnd1 is absent from the same evolutionary lineages that lack Dmc1 [84], we show that Hop2-Mnd1 is specifically required for Dmc1-mediated strand exchange in budding yeast meiosis and does not facilitate or modulate Rad51-dependent strand exchange *in vivo*. Moreover, Hop2-Mnd1 is required even when Dmc1 catalyzes strand exchange between sister chromatids [85].

### Hed1 promotes interhomolog bias and SC morphogenesis, and mitigates the effects of suboptimal DSB levels

*In vitro* and *in vivo* studies provide clear evidence that Hed1 inhibits Rad51's strand exchange activity, which blocks meiotic DSB-repair when Dmc1 is absent [47], [48], [86]. However, the physiological role of Hed1 during unperturbed meiosis has remained uncertain. Our analysis reveals that Hed1 facilitates Dmc1-dependent interhomolog bias, the efficient incorporation of Zip1 into SCs, and the organization of alternating domains of Red1 (+Hop1) and Zip1 (+Rec8) protein abundance by limiting Red1 loading (Figures 2, 5 and 10A). The most straightforward interpretation of Hed1's role in promoting homolog bias is that it is solely a consequence of its ability to inhibit the strand exchange activity of Rad51. However, it is also possible that Hed1 modulates the Rad51 ensemble in a positive way to help promote interhomolog partner choice via Dmc1-mediated strand-exchange. In this context, it is worth noting that Hed1 has been shown to stabilize Rad51 filaments [86]. Such stabilization could, in principle, enhance the regulatory roles of Rad51 during meiosis.

A *hed1* mutation also renders cells unable to compensate for modest reductions in DSB levels, indicating that Hed1-dependent interhomolog bias is especially important in cells with suboptimal DSB levels. These data also raise the possibility that the ∼5-fold interhomolog bias seen in wild-type cells reflects a physiologically important “set point” that enables individual cells to compensate for stochastic variation in DSB numbers. Finally, we note that the phenotypes conferred by *hed1* mutation are similar to those of cells lacking the AAA+ ATPase, Pch2, and may reflect a common defect in enforcing interhomolog template choice [73], [74], [87].

### Hed1 and Mek1 make independent contributions to interhomolog bias

Together with previous studies, our data indicate that the concerted action of at least four components promote interhomolog bias catalyzed by Dmc1-dependent DNA strand exchange (summarized in Figure 10A): (i) a strand exchange-independent activity of Rad51 and its accessory factors [32], [46]; (ii) phospho-kinase signaling via the Red1-Hop1-Mek1 pathway [32], [88], [89]; (iii) inhibition of Rad51-dependent strand exchange by Hed1; and (iv) inhibition of Rad51-dependent strand exchange by Dmc1.

When Dmc1 is absent, both Hed1 and Mek1 are required to inhibit Rad51-dependent recombination, which occurs primarily between sister chromatids [32], [66], [67]. Thus, one aspect of Mek1's role in homolog bias is inferred to be inhibition of Rad51-catalyzed inter-sister strand exchange [43], [90]. However, once Dmc1 is incorporated into the recombination complex, interhomolog bias is largely independent of Hed1 (Figure 2; Figure 3).

The above considerations imply that the relatively strong interhomolog bias defect seen in *mek1* mutants compared to *hed1* mutants is not explained by Rad51 inhibition alone. Moreover, in mutants lacking factors required for assembly of Rad51 filaments (mediator complexes Rad55-Rad57 and Psy3-Csm2-Shu1-Shu2, as well as Rad51 itself; [32], [85]) Dmc1 catalyzes recombination between sister-chromatids indicating that the interhomolog bias function of Mek1 requires the presence of a complete Rad51 ensemble. These considerations invoke a model in which Mek1's kinase activity cooperates with Rad51 complexes to direct Dmc1-dependent strand exchange between homologs (Figure 10). In this context, we note that Mek1 and Rad51 are also proposed to play positive roles in homolog bias by locally removing inhibitory effects of sister chromatid cohesion on formation on interhomolog interactions [44].

Tsubouchi and Roeder [47] suggested that one role of Hed1 may be to inhibit Rad51 while Dmc1 filaments are assembled, such that the activities of Dmc1 and Rad51 are coordinated. Our data are in agreement with this proposal and further suggest that the requirement for Hed1 in Rad51 inhibition is largely alleviated once Dmc1 filaments are assembled. The ability of Dmc1 to inhibit Rad51-mediated intersister recombination also suggests a possible explanation for how organisms that lack obvious Hed1 orthologs might limit Rad51-mediated inter-sister recombination; the transcriptional programs in such organisms may ensure induction of sufficiently high Dmc1 levels prior to DSB formation such that Rad51 can be immediately inhibited as recombinosomes assemble.

### Dmc1 promotes synapsis and normal morphogenesis of axial/lateral elements

Although elongated SCs can form in *dmc1 hed1* cells, their formation is inefficient and the composition of these structures is highly abnormal. Most notably, the density of Red1 along lateral elements is significantly lower in *dmc1 hed1* than in wild type (Figure 5). Thus, a Dmc1-dependent process promotes assembly or stabilization of Red1 in local abundance domains along the SCs. It is possible that Dmc1 influences Red1 localization prior to synapsis, although technical limitations prevent this possibility from being tested at present. If Dmc1 does promote Red1 localization prior to synapsis, this activity will concentrate Red1 around developing recombinosomes, which will locally enhance Mek1-kinase activity and thereby enhance inter-homolog bias (Figure 10A).

### Dmc1-dependent interhomolog bias is important for crossover control

Although previous analyses showed that high levels of crossing-over occur when Rad51 is activated in the absence of Dmc1 [10], [45], these studies could not assess the efficiency of crossover assurance. Here we show that despite near wild-type frequencies of crossing-over in the cell population, crossover assurance is very inefficient in *dmc1 hed1* cells. This defect is most readily explained by the stochastic failure of homologs to stably pair due to insufficient interhomolog interactions. In this regard, we can infer that Dmc1-dependent interhomolog bias is an essential precondition for efficient crossover assurance.

We note that although chromosome III fails to crossover in ∼15% of *dmc1 hed1* cells, nondisjunction of this chromosome is detected in only 1.5% of tetrads, much lower than the ∼7.5% expected for random segregation of achiasmate homologs. It can be inferred that the well-characterized back-up segregation system [91], [92], [93], [94] makes a major contribution to accurate achiasmate disjunction and spore viability in *dmc1 hed1* cells, further belying the severity of the recombination defect in this strain.

Published studies indicated that crossover interference is diminished when Rad51 catalyzes meiotic recombination [10], [45]. However, population heterogeneity could have contributed to the apparent reduction in interference in these experiments [95]. Specifically, cell-to-cell heterogeneity in recombination activity may have arisen from variable copy number of the *RAD51* and *RAD54* plasmids used to activate Rad51-dependent recombination in these studies. Moreover, defective crossover assurance will exacerbate population heterogeneity and dilute interference signals ([76]; Figure S7). Our analysis allows these effects to be separated to more definitively assess interference between Rad51-dependent crossovers. Although interference is significantly weakened in *dmc1 hed1* cells, we can conclude that the mechanisms responsible for interference retain significant activity.

Reduced interference could arise from changes caused by defective interhomolog bias in *dmc1 hed1* cells. One possibility is that adjacent recombinational interactions arise less synchronously in *dmc1 hed1* cells than in wild type (discussed further below). Early arising recombination events may not interfere with late events (and *vice versa*) resulting in dilution of the overall intensity of crossover interference. In support of this idea, delayed, asynchronous, and inefficient chromosome pairing can perturb interference in *C. elegans* [96], [97].

### Feedback processes buffer against defective interhomolog bias

In *dmc1 hed1* cells, 5-fold defects in template choice are observed at the *HIS4::LEU2* and *ERG1* hotspots (Figure 2), but final crossover levels at these loci are reduced by only 3.5- and 2.1-fold, respectively (Figure 1). Moreover, the chromosome-wide crossover frequency is only marginally lower than wild type (1.1 fold reduction; Figure 6). Our physical and genetic analyses indicate that feedback processes respond specifically to low levels of interhomolog interactions to enhance processes that eventually lead to formation near normal crossover frequencies (Figure 10B). Two distinct feedback processes are indicated:

#### (i) Enhanced crossover bias

Keeney and colleagues first demonstrated that the outcome of meiotic DSB-repair is modulated to favor crossing-over when global DSB levels are reduced [18]. Two observations imply that crossover bias is already operating at maximal capacity in *dmc1 hed1* cells: first, the crossover/non-crossover ratio is already much higher than seen for the most severe combination of *SPO11* alleles (*spo11-HA*/*spo11-yf*, 30% of wild-type DSB levels); and second, little or no additional increase in the crossover/non-crossover ratio is observed when global DSB levels are reduced (Figure 8). These observations support our inference that the pertinent metric for this aspect of crossover homeostasis is the number of interhomolog interactions, i.e. reduced interhomolog bias effectively mimics reduced DSB levels. Moreover, analysis of recombinants at *ERG1* also indicate that this crossover bias process is capable of converting interhomolog events into crossovers with >80% efficiency.

#### (ii) Further activation of recombination

Given the severity of the interhomolog bias defect seen at DSB hotspots in *dmc1 hed1* cells, the degree of crossover outcome bias detected at these sites is insufficient to account for the final crossover levels (crossover bias reproducibly accounts for only ∼20–50% of apparent compensation for homolog bias defects; see Text S1 for supplementary discussion and calculations). Assuming that the interhomolog bias defect measured at DSB hotspots is representative of all sites, even absolute crossover bias cannot account for the high global crossover levels seen in *dmc1 hed1* cells.

We infer that crossover bias cannot be the sole mechanism used to compensate for the interhomolog bias defect of *dmc1 hed1* cells. We propose that additional DSBs are formed in *dmc1 hed1* cells. Specifically, we suggest that additional DSBs form as a response to delayed chromosome pairing or synapsis. Consistent with this idea, recent analysis of meiosis in mouse spermatocytes indicates that additional DSBs form in regions of chromosomes that fail to synapse [98].

The different effects of *dmc1 hed1* mutation on crossover levels measured at DSB hotspots compared to larger chromosomal intervals (e.g. Figure 9), is explained by the likelihood that physically large regions represent large reserves of potential DSBs. As such, larger regions have a greater capacity for deferred activation of DSB formation and, ultimately, for crossover homeostasis. Conversely, in a very short interval, potential DSB sites will be few and local potential for homeostasis will be primarily dependent on the crossover bias process. An extreme example of short versus long-range effects is seen when comparing the 4.3 kb region surrounding the *HIS4::LEU2* DSB site with the adjacent 130 kb interval (Figure 9).

Additional DSB formation could occur as a relatively passive consequence of the fact that DSB formation is not completely shutdown until Ndt80 is expressed [29], [30], [99]. In *dmc1 hed1* cells, the extended prophase caused by delayed DSB-repair will provide a longer time window for DSB formation. In addition to this pan-nuclear process, a feedback mechanism may promote continued DSB formation; and conversely, synapsis may generally shutdown DSB formation on a per chromosome basis [98], [100].

Physiological causes of delayed homolog pairing and synapsis include low DSB numbers, reduced interhomolog bias, chromosome entanglements and non-allelic interactions. We conclude that the two compensatory feedback pathways, described above, comprise a robust and efficient global crossover homeostasis process that can compensate for stochastic cell-to-cell variation in the efficiency of homolog pairing and synapsis, regardless of its root cause. Mechanistic understanding of these feedback processes is an important goal for the future.

During the preparation of this manuscript we learned that Kim and colleagues also demonstrated that *hed1* mutants display weak, and *dmc1 hed1* mutants strong, interhomolog bias defects at the *HIS4::LEU2* locus. Those data were published recently [101].

## Materials and Methods

### Yeast strains

Strain information is listed in Supplemental Table S6. All strains are derivative of strain SK-1.

### Meiotic time course

Synchronous yeast cultures were induced to undergo meiosis by transfer to sporulation media as described previously [4]. Samples were taken over time to monitor the events of meiotic recombination and SC assembly. Meiotic progression was monitored by counting the number of DAPI staining bodies per cell for at least 100 cells per time point.

### Tetrad analysis

Haploid strains were mated for 4–24 hrs on YPD plates and sporulated on plates containing 1% potassium acetate and 0.02% raffinose at 30°C for 2 days. Alternatively, asci were taken from meiotic time course after 48 hrs. Asci were digested with zymolyase and dissected on YPD plates supplemented with adenine, uracil, methionine, lysine, and threonine. Only tetrads producing four viable spores and showing Mendelian segregation of markers were used to calculate genetic map distance. Map distances were determined using the Perkins equation: (100(6NPD+TT))/(2(PD+NPD+TT)) [102]. Standard errors were calculated using Stahl Lab Online Tools (http://www.molbio.uoregon.edu/~fstahl/). The NPD ratio for interference analysis is calculated as the fraction of NPDs observed/fraction of NPDs expected. NPD expected is calculated under the assumption where there is no interference using the Papazian equation: NPD expected = 0.5[(1-fT)−(1-(3fT/2))^2/3^], where fT denotes the observed frequency of tetratypes [77].

### Construction of a system to monitor JMs at a native DSB hotspot

The diploid strain used for JM analysis at *ERG1* was engineered by a series of two-step gene replacements. On one copy of chromosome *VII, SacII* restriction sites were engineered into intragenic regions at *Saccharomyces* Genome Database (SGD) (S288c genome assembly) coordinate 844276 (*YGR173w*) and coordinate 854464 (*YGR179c*); and a *Sal*I site was engineered between *ERG1* and *YGR177c* at coordinate 848724. For the *Sac*II site at coordinate 844276 the region between coordinates 843173 and 845291 was PCR amplified (5′ and 3′ primers contain *Hind*III restriction sites), digested with *Hind*III, and cloned into pRS306. The *Sac*II site was introduced by Quickchange Site-Directed Mutagenesis (Invitrogen) with the primers 5′-GTTGTTGCCACAGCAAGGACCGCGGATCTAGTATTAATGG and 5′-CCATTAATACTAGATCCGCGGTCCTTGCTGTGGCAACAAC (A→G). This plasmid was subsequently linearized using a unique *Msc*I restriction site. For the *Sac*II site at coordinate 854464, the region between coordinates 853473 and 855252 was PCR amplified (5′ and 3′ primers contain *Hind*III restriction sites), digested with *Hind*III, and cloned into pRS306. The *Sac*II site was introduced by site-directed mutagenesis with the primers 5′-GCTTTAATTCATAATTCCGCGGCAACCTTTCTCTATACTCAGC and 5′-GCTGAGTATAGAGAAAGGTTGCCGCGGAATTATGAATTAAAGC (G→C). This plasmid was linearized using a unique *EcoN*I restriction site. For the *Sal*I restriction site at coordinate 848724, the region between coordinates 847679 and 849776 was PCR amplified (5′ and 3′ primers contain *Hind*III restriction sites), digested with *Hind*III, and cloned into pRS306. The *Sal*I site was introduced by site-directed mutagenesis with the primers 5′-GCAGCCACGGCATGCGTCGACTACGAGCGTATTGTG and 5′-CACAATACGCTCGTAGTCGACGCATGCCGTGGCTGC (A→G). This plasmid was linearized using a unique *Age*I restriction site.

On the other copy of chromosome *VII, SacII* restriction sites were engineered at SGD coordinate 845470 (intergenic) and coordinate 852145 (*YGR178c*) and a *Spe*I site was engineered between *ERG1* and *YGR177c* at coordinate 848683. For the *Sac*II restriction site at coordinate 845470, the region between coordinates 844520 and 846322 was PCR amplified (5′ and 3′ primers contain *Hind*III restriction sites), digested with *Hind*III, and cloned into pRS306. The *Sac*II site was introduced by site-directed mutagenesis with the primers 5′-GGTTTAGATCCAAGATTCCGCGGTTCCACCATTTAATATG and 5′-CATATTAAATGGTGGAACCGCGGAATCTTGGATCTAAACC (C→G). This plasmid was linearized using a unique *Nru*I restriction site. For the *Sac*II restriction site at coordinate 852145, the region between coordinates 851037 and 853268 was PCR amplified (5′ and 3′ primers contain *Sal*I restriction sites), digested with *Sal*I, and cloned into pRS306. The *Sac*II site was introduced by site-directed mutagenesis with the primers 5′-CCTCTGGCGCACCTGCTGCCGCGGGAGTAGAGGTATCCG and 5′-CGGATACCTCTACTCCCGCGGCAGCAGGTGCGCCAGAGG (T→C). This plasmid was linearized using a unique *Bgl*II restriction site. For the *Spe*I restriction site at coordinate 848683, the region between coordinates 847679 and 849776 was PCR amplified (5′ and 3′ primers contain *Hind*III restriction sites), digested with *Hind*III, and cloned into pRS306. The *Spe*I site was introduced by site-directed mutagenesis with the primers 5′-CATGCGAGGTAAGACTAGTGTCTGAGACTTATACCCGACC and 5′-GGTCGGGTATAAGTCTCAGACACTAGTCTTACCTCGCATG (T→G). This plasmid was linearized using a unique *Age*I restriction site.

### DNA physical assays

Recombination events at *HIS4::LEU2* and *ERG1* loci were similarly monitored by gel electrophoresis. The *HIS4::LEU2* assay system contains *XhoI* restriction site polymorphisms between parental homologs producing fragments diagnostic for parental and recombinant chromosomes. A *BamHI/NgoMIV* polymorphism immediately at the DSB site allows detection of non-crossover products. Non-crossovers were analyzed by double digestion of genomic DNA with *XhoI* and *NgoMIV* and separation on one-dimensional gels. The *ERG1* locus contains *Sac*II restriction site polymorphisms that produce fragments diagnostic for JMs and recombinant products. To analyze relative amounts of crossover and non-crossover products at *ERG1*, genomic DNA was doubly digested with *Sac*II and *Sal*I and analyzed by one-dimensional gel and Southern hybridization. The polymorphic *Sal*I site located at the DSB site is diagnostic for non-crossover products. Samples were treated with psoralen and UV to crosslink DNA and stabilize JM intermediates using previously described methods [36], [54], [103]. DNA extraction and Southern blot analysis were carried out using methods that have been described in detail previously [100].

### Yeast cytology and chromosome spreads for immunofluorescence

Yeast cells were fixed and spread as described previously [4], [103]. For STED microscopy ProLong Gold (Invitrogen Molecular Probes, catalog # P36930) was used as an anti-fade reagent instead of vectashield. Spreads were stained with primary antibodies followed by incubation with fluorochrome-conjugated secondary antibodies (Invitrogen Molecular Probes, 1∶1000 dilution). Images were acquired using Zeiss Axiovision 4.6 at 100×magnification. For STED microscopy images were acquired using a Leica SP5 II STED-CW at 100×magnification. Images were adjusted for brightness and contrast using NIH ImageJ. NIH ImageJ was utilized to measure intensities of staining structures. The rabbit anti-Ctf19 antibody was a gift from Phil Heiter and was used following 1 to 1000 dilution. The rabbit anti-Zip3 and rabbit anti-Rec8 antibodies were gifts from Akira Shinohara and were both used diluted 1∶500. The rabbit anti-Red1 antibody used was a gift from Shirleen Roeder and was used at a 1∶500 dilution. The Goat anti-Zip1 antibody is commercially available via Santa Cruz (catalog # sc-15632).

### Random spore analysis to test crossover homeostasis

Random spore analysis was performed as described [18]. Briefly, strains were induced to undergo sporulation, harvested, and asci were digested with zymolyase, diluted in 0.1% Tween-20, sonicated to produce single spores, and plated onto solid media lacking arginine. *ARG+* colonies were then tested for growth on plates lacking uracil and threonine.

### Visualization of the SC via electron microscopy

Nuclei were first fixed and spread followed by silver staining to visualize the synaptonemal complex [104], [105]. To prepare spheroplasts, 10 ml of meiotic culture was collected and spun for 2.5 min at 1,600 rpm, the pellet was resuspended in 2 ml of ZK buffer (25 mM Tris pH 7.5, 0.8 M KCl), and 40 µl of 1 M DTT was added. After a 2 min incubation at room temperature, cells were spun for 2.5 min at 1,600 rpm, the pellet was then resuspended in 2 ml ZK buffer, 15 µl zymolyase solution (50 mM Tris pH 7.5, 2% glucose, 20 mg/ml zymolyase) was added, and cells were incubated for 20 min with gentle rotation at 30°C. Cells were then spun for 2.5 min at 1,300 rpm, washed in 5 ml MES (1 M sorbitol, 0.1 M MES pH 6.5, 1 mM EDTA, 0.5 mM MgCl_2_), and pelleted as before. Next, cells were resuspended in 1 ml MES and spun for 1 min at 200 rpm, followed by aspiration of the supernatant. To release nuclei from the spheroplasts, 5 µl of the pellet was placed into 50 µl MEM/protease inhibitor (0.1 M MES pH 6.8, 1 mM EDTA, 0.5 mM MgCl_2_) with freshly added 0.1 M PMSF (10 µl of PMSF stock to 1 ml MEM), then gently resuspended by pipetting. To fix the released nuclei, 50 µl of PFA was added and mixed well. 50 µl of the final mixture was spread across microscope slide precoated with polystyrene plastic and incubated at room temperature in a humid chamber. After 5 min, an additional 400 µl of PFA was pipetted onto the slide. After 5 min more, preparations were rinsed with 4 ml of 0.4% Photoflo (Kodak) and allowed to dry completely before proceeding to silver staining.

### Silver staining

200 µl of colloidal developer and 200 µl silver nitrate were mixed on a 24×50 coverslip and the mixture was put on top of a slide that had fixed nuclei. The preparation was placed on a 60°C hotplate for 2–5 min, then gently rinsed with water to remove the coverslip and stop the reaction. Once dry the polystyrene membrane was floated off on a water surface and clean EM grids were placed on the membrane. The membrane and grids were removed from H_2_O and allowed to dry. Images were taken at 3,500×magnification using a Hitachi H-7600 electron microscope housed in the Oklahoma Medical Research Foundation Core Facility for Imaging.

## Supporting Information

Figure S1 Independent analysis of JM formation and crossing-over in *hed1, dmc1*, and *dmc1 hed1* strains. A. Representative Southern images of JMs resolved by 2D gels. B. Timing and efficiency of meiotic divisions. C. Quantification of JMs over time. D. IH/IS dHJ ratios over time. E. Images of 1D Southern analysis of crossing-over at the *HIS4::LEU2* locus. F. Quantification of final crossover levels at the *HIS4::LEU2*. Averages of four independent time courses are shown. Error bars show standard error.
(TIFF)

Figure S2 Analysis of JMs in the *ndt80* background. A. 2D Southern images showing accumulated JMs in *ndt80, hed1 ndt80* and *dmc1 hed1 ndt80* strains. B. IH/IS dHJ ratios at 7 and 8 hrs after induction of meiosis. C. Quantification of various JM species in *ndt80* strains. *Note that the SEI-like species are distinct from the SEIs that form at early times, which form more discrete signals and have a defined strand composition [52]. However, the exact nature of these structures and their fate remain unclear.
(TIFF)

Figure S3 *rad51* is epistatic to *hed1*. A. Representative Southern images showing 1D gel analysis of crossing over at *HIS4::LEU2* in *rad51* and *rad51 hed1* time course experiments. B. Quantitation of crossovers at 12 and 24 hours in *rad51* and *rad51 hed1* cells. Each strain was analyzed in triplicate. C. Representative Southern images of 2D gels showing JM analysis at *HIS4::LEU2* in wild-type, *rad51* and *rad51 hed1* strains. Lower panels show blowups of the JM regions. D. Quantification of IH-dHJs and IS-dHJs at *HIS4::LEU2* in wild-type, *rad51* and *rad51 hed1* strains. E. Southern images showing 1D gel analysis of DSB formation and crossing over at *HIS4::LEU2* in wild-type, *dmc1 rad51* and *dmc1 rad51 hed1* time course experiments. F. Quantification of the Southerns shown in E. The apparent reduction of DSB signals at late times in *dmc1 rad51* and *dmc1 rad51 hed1* strains results from excessive resection of DSBs past the diagnostic *Xho*I restriction site. Residual crossover products detected in *rad51 dmc1* double mutants (∼10–25% of normal crossover levels) have been noted previously [47], [81]. It is unclear whether these products represent *bond fide* reciprocal crossovers. The aberrant pathway responsible for these products has not been defined, but JMs are not detected in the *rad51 dmc1* double mutant [32], [106].
(TIFF)

Figure S4 JM analysis in *mek1-as* strains. A. Representative Southern images of 2D gels showing JM analysis at *HIS4::LEU2* in wild-type, *mek1-as*, *hed1*, and *mek1-as hed1* cells. Lower panels show blowups of the JM regions. B. Timing and efficiency of meiotic divisions in wild-type, *hed1*, *mek1-as*, and *mek1-as hed1* cells. C. Quantification of IH-dHJs, IS-dHJs and the IH/IS dHJ ratio in wild-type, *mek1-as*, *hed1*, and *mek1-as hed1* cells.
(TIFF)

Figure S5 Additional images of Red1 immunostaining analyzed by STED microscopy. Red1 (red) is imaged via STED and Zip1 by confocal microscopy.
(TIFF)

Figure S6 Immunostaining and EM analysis of spread meiotic nuclei shows that elongated Zip1 structures formed in the *dmc1 hed1* mutant are SCs. A. Representative immunostained nuclei showing Zip1 (green), Zip3 (red) and merged channels. Note that Zip3 foci colocalize with elongated ZIp1 structures. B. Average numbers of Zip3 foci per nucleus for wild-type, *hed1*, *dmc1 hed1*, and *dmc1* cells. At lest 50 nuclei were analyzed for each strain. C. Electron micrographs of SCs stained with silver. Both wild-type nuclei and one of the *dmc1 hed1* nuclei have fully formed tripratite SCs.The *hed1 dmc1* nucleus to the left displays regions of tripartite SC as well as unsynapsed regions.
(TIFF)

Figure S7 Analysis of crossover interference along chromosome III with and without non-exchange tetrads. A. NPD ratios. In the graph on the left, NPD ratio were calculated for the *dmc1 hed1* dataset that included non-exchange tetrads. In the graph on the right, non-exchange tetrads were excluded from the *dmc1 hed1* dataset. The asterisks indicate intervals in *dmc1 hed1* dataset that show a significant change from wild type. B. Analysis of interference using the “adjacent intervals” approach of Malkova et al. [76]. Ratios of map distances Adj^CO^/Adj^PD^ for wild-type, *hed1*, and *dmc1 hed1* are shown. The reported ratios are averages of the two ratios for each interval pair. Solid lines represent significant interference and dashed lines represent non-significant deviations from a ratio of one. The panels on the left shows the analysis for *dmc1 hed1* with non-exchange tetrads included and the panel on the right shows the analysis for *dmc1 hed1* with non-exchange tetrads excluded. The asterisks indicate intervals with a significant change from wild type.
(TIFF)

Figure S8 Analysis of crossover interference for distant intervals. Analysis of interference between distant intervals using the nearest neighbor approach of Malkova et al. (2004). Ratios of map distances Adj^CO^/Adj^PD^ for wild type, *dmc1 hed1* including non-exchange tetrads, and *dmc1 hed1* excluding non-exchange tetrad. The number is the average of the two Adj^CO^/Adj^PD^ ratios obtained by taking each interval as reference. Solid lines represent significant interference and dotted lines represent non-significant interference. *dmc1 hed1* shows significant negative interference when non-exchange tetrads are included in the analysis.
(TIFF)

Table S1 Quantitation of Red1, Zip1, and Zip3 localization.
(PDF)

Table S2 Quantitation and comparison of Red1 staining by STED microscopy.
(PDF)

Table S3 Analysis of crossover interference using non-parental ditype ratios.
(PDF)

Table S4 Analysis of crossover interference using adjacent intervals.
(PDF)

Table S5 Analysis of crossover interference for distant intervals.
(PDF)

Table S6 Yeast strains used in this study.
(PDF)

Text S1 Supplementary discussion.
(PDF)
