# Supplementary figures and images for: Meiotic Crossover Control by Concerted Action of Rad51-Dmc1 in Homolog Template Bias and Robust Homeostatic Regulation
Source: PLoS Genet. 2013 Dec 19;9(12):e1003978. doi: 10.1371/journal.pgen.1003978 (PMC3868528; doi:10.1371/journal.pgen.1003978)

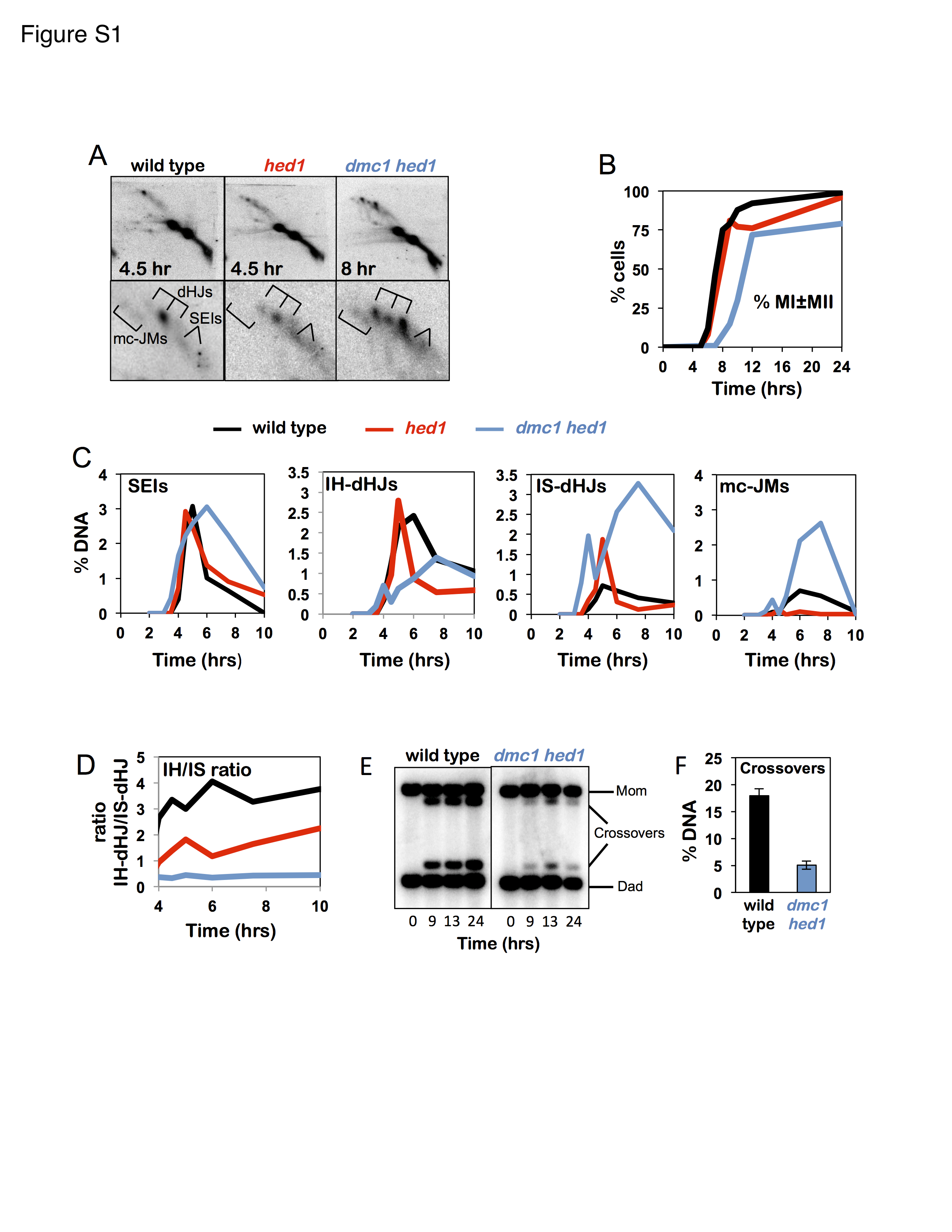

Supplement: Figure S1 — Independent analysis of JM formation and crossing-over in hed1, dmc1, and dmc1 hed1 strains. A. Representative Southern images of JMs resolved by 2D gels. B. Timing and efficiency of meiotic divisions. C. Quantification of JMs over time. D. IH/IS dHJ ratios over time. E. Images of 1D Southern analysis of crossing-over at the HIS4::LEU2 locus. F. Quantification of final crossover levels at the HIS4::LEU2. Averages of four independent time courses are shown. Error bars show standard error. (TIFF) [file pgen.1003978.s001.tiff]

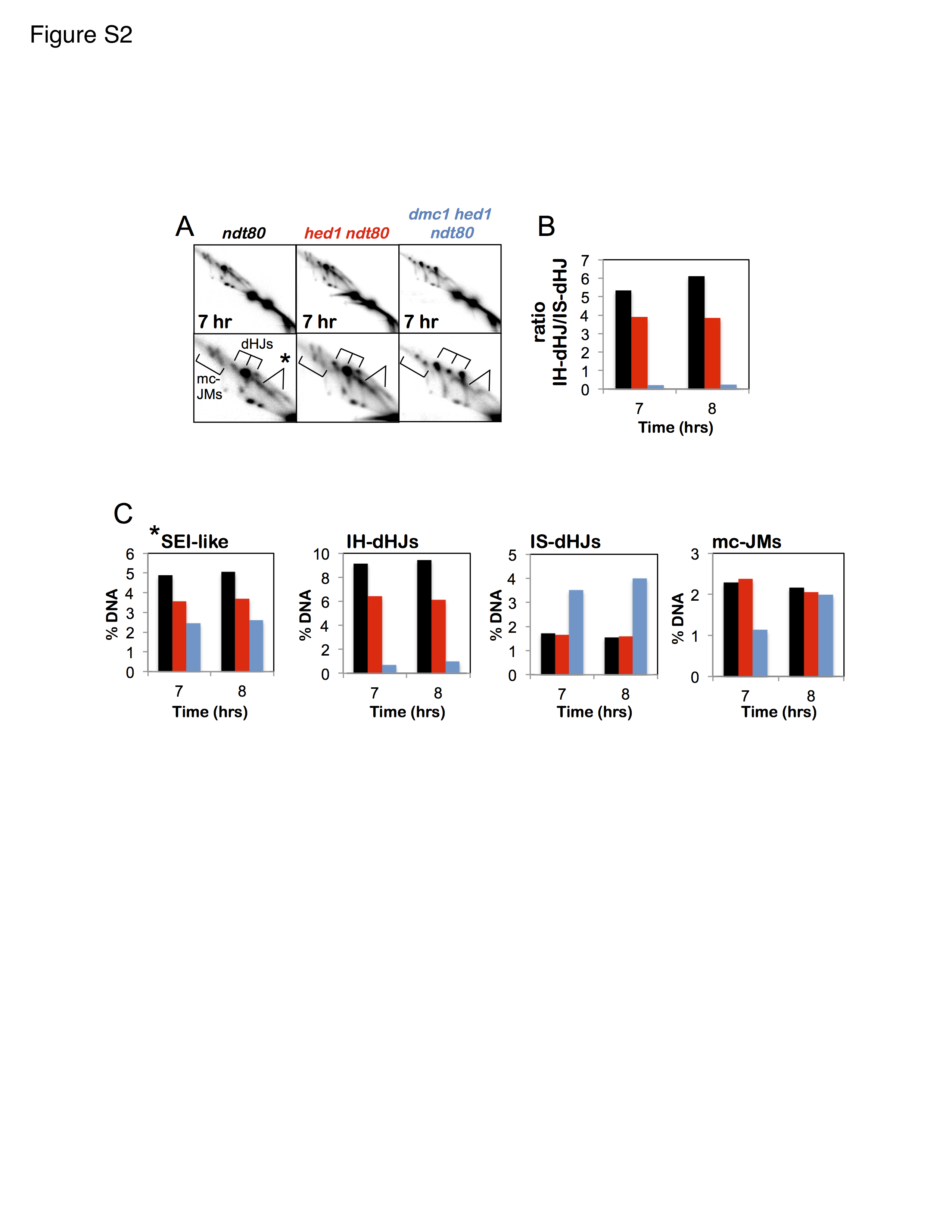

Supplement: Figure S2 — Analysis of JMs in the ndt80 background. A. 2D Southern images showing accumulated JMs in ndt80, hed1 ndt80 and dmc1 hed1 ndt80 strains. B. IH/IS dHJ ratios at 7 and 8 hrs after induction of meiosis. C. Quantification of various JM species in ndt80 strains. *Note that the SEI-like species are distinct from the SEIs that form at early times, which form more discrete signals and have a defined strand composition [52]. However, the exact nature of these structures and their fate remain unclear. (TIFF) [file pgen.1003978.s002.tiff]

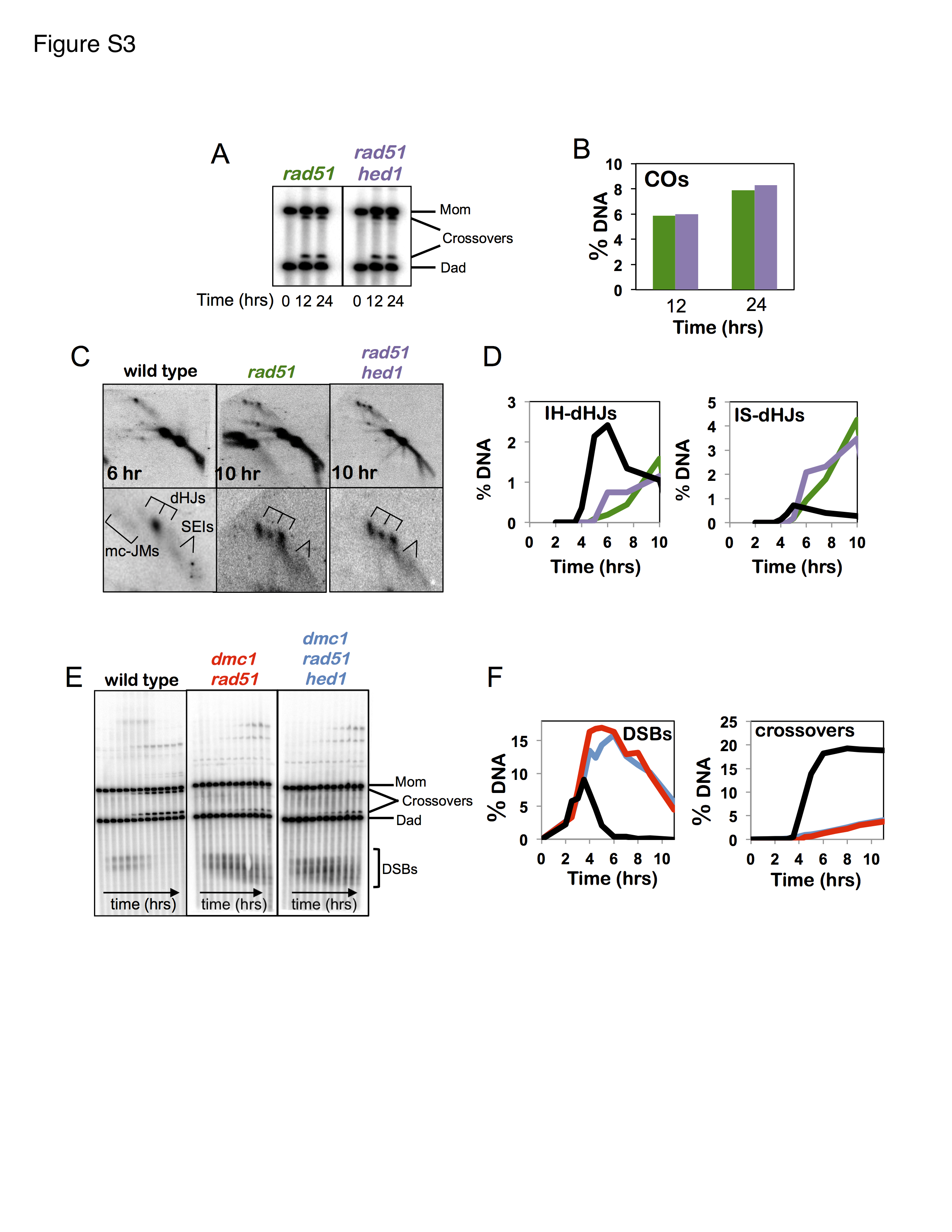

Supplement: Figure S3 — rad51 is epistatic to hed1. A. Representative Southern images showing 1D gel analysis of crossing over at HIS4::LEU2 in rad51 and rad51 hed1 time course experiments. B. Quantitation of crossovers at 12 and 24 hours in rad51 and rad51 hed1 cells. Each strain was analyzed in triplicate. C. Representative Southern images of 2D gels showing JM analysis at HIS4::LEU2 in wild-type, rad51 and rad51 hed1 strains. Lower panels show blowups of the JM regions. D. Quantification of IH-dHJs and IS-dHJs at HIS4::LEU2 in wild-type, rad51 and rad51 hed1 strains. E. Southern images showing 1D gel analysis of DSB formation and crossing over at HIS4::LEU2 in wild-type, dmc1 rad51 and dmc1 rad51 hed1 time course experiments. F. Quantification of the Southerns shown in E. The apparent reduction of DSB signals at late times in dmc1 rad51 and dmc1 rad51 hed1 strains results from excessive resection of DSBs past the diagnostic XhoI restriction site. Residual crossover products detected in rad51 dmc1 double mutants (∼10–25% of normal crossover levels) have been noted previously [47], [81]. It is unclear whether these products represent bond fide reciprocal crossovers. The aberrant pathway responsible for these products has not been defined, but JMs are not detected in the rad51 dmc1 double mutant [32], [106]. (TIFF) [file pgen.1003978.s003.tiff]

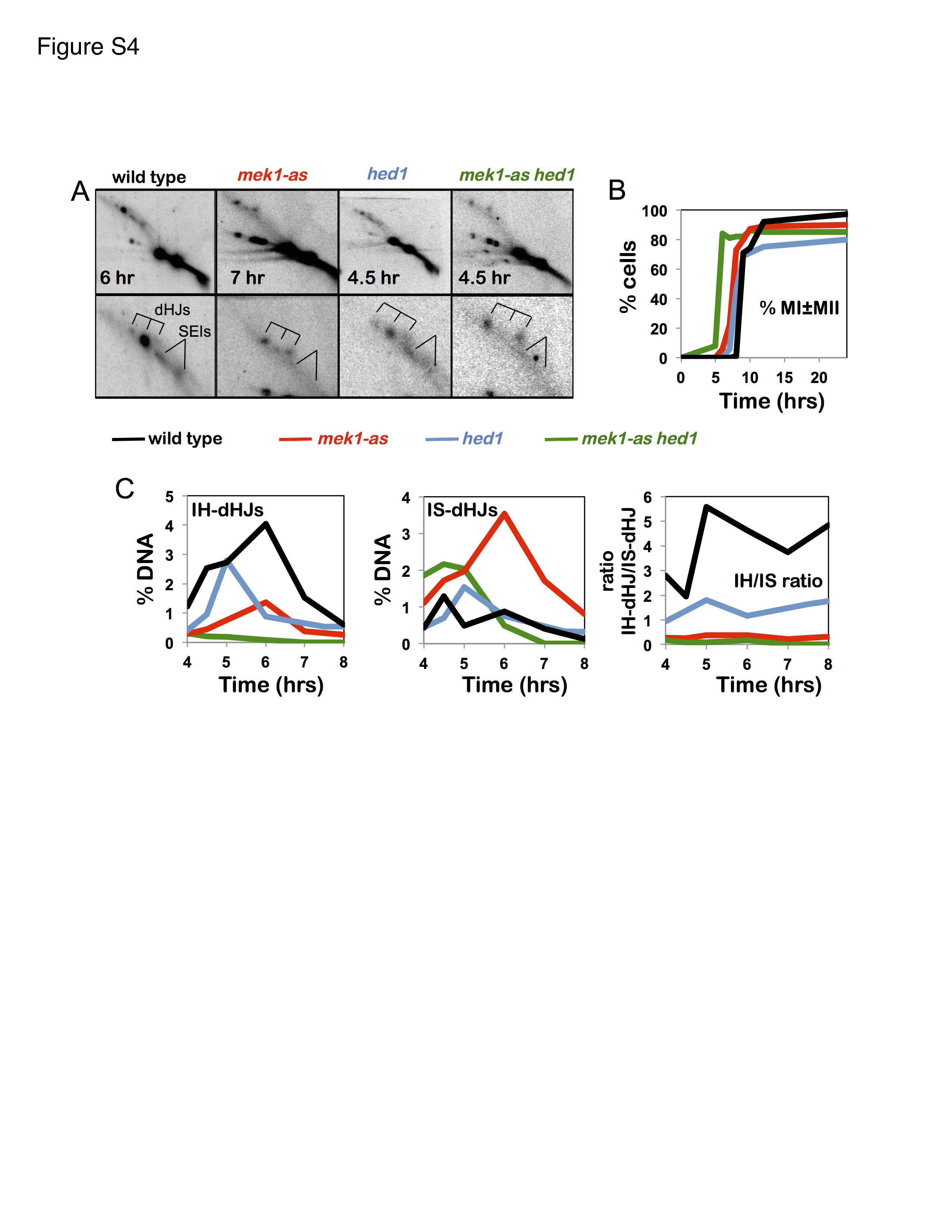

Supplement: Figure S4 — JM analysis in mek1-as strains. A. Representative Southern images of 2D gels showing JM analysis at HIS4::LEU2 in wild-type, mek1-as, hed1, and mek1-as hed1 cells. Lower panels show blowups of the JM regions. B. Timing and efficiency of meiotic divisions in wild-type, hed1, mek1-as, and mek1-as hed1 cells. C. Quantification of IH-dHJs, IS-dHJs and the IH/IS dHJ ratio in wild-type, mek1-as, hed1, and mek1-as hed1 cells. (TIFF) [file pgen.1003978.s004.tiff]

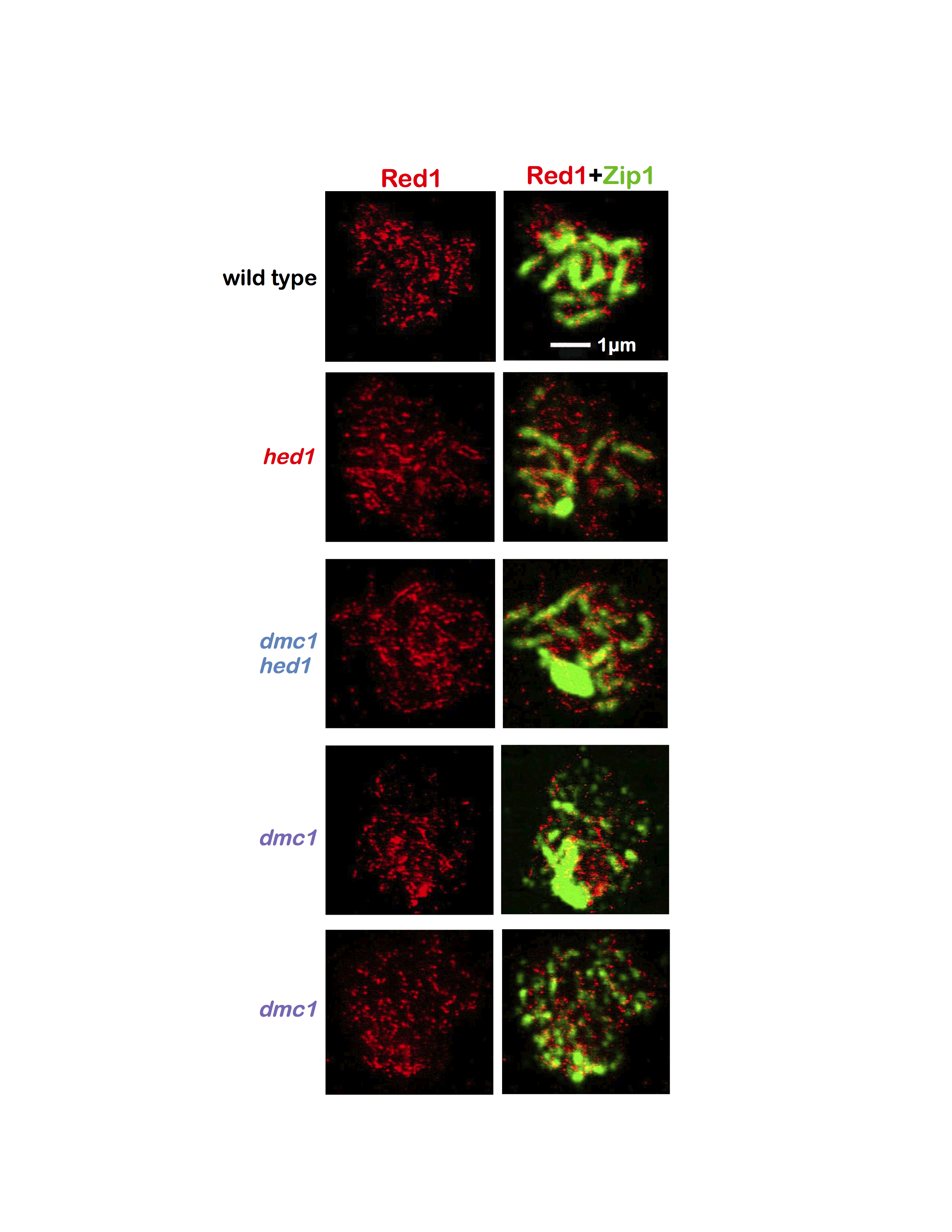

Supplement: Figure S5 — Additional images of Red1 immunostaining analyzed by STED microscopy. Red1 (red) is imaged via STED and Zip1 by confocal microscopy. (TIFF) [file pgen.1003978.s005.tiff]

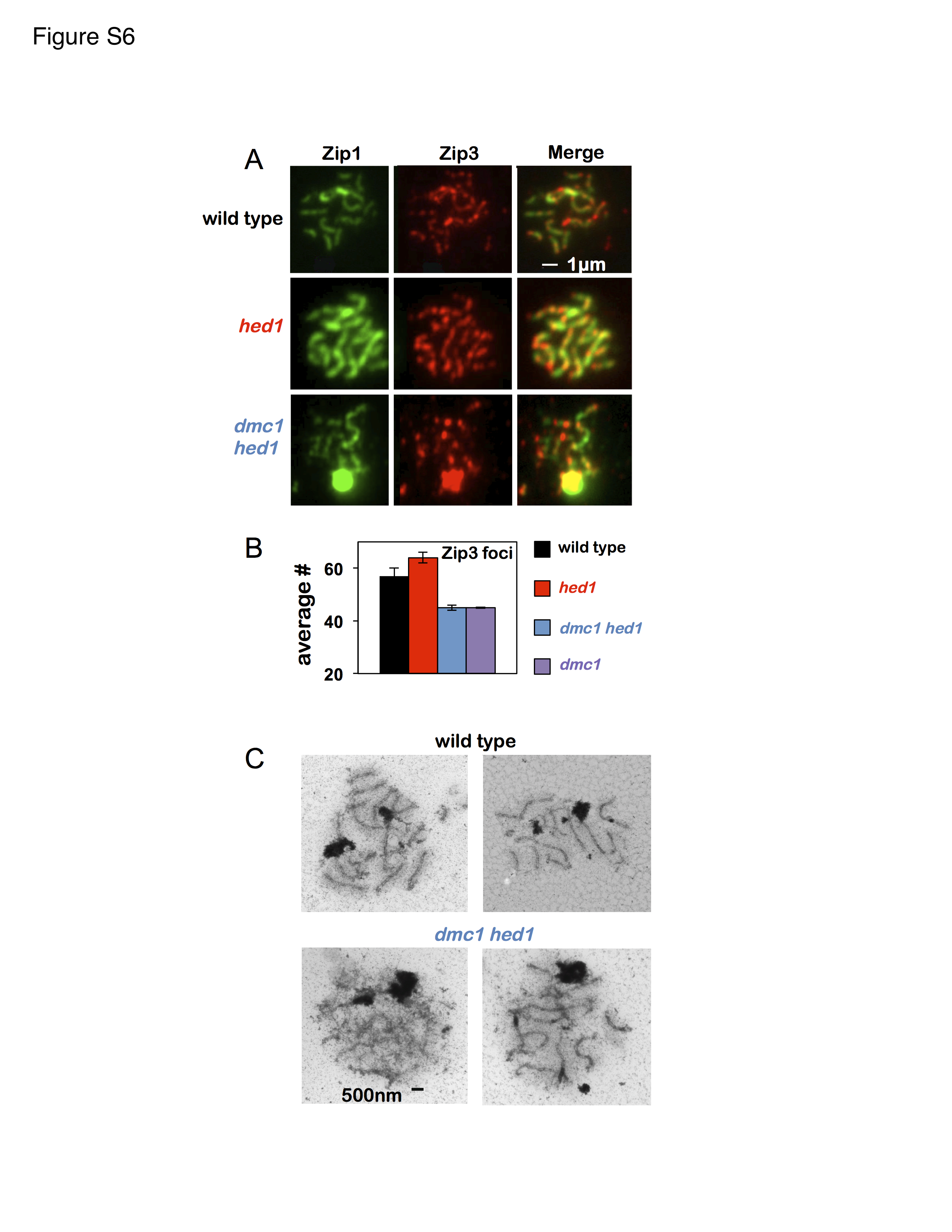

Supplement: Figure S6 — Immunostaining and EM analysis of spread meiotic nuclei shows that elongated Zip1 structures formed in the dmc1 hed1 mutant are SCs. A. Representative immunostained nuclei showing Zip1 (green), Zip3 (red) and merged channels. Note that Zip3 foci colocalize with elongated ZIp1 structures. B. Average numbers of Zip3 foci per nucleus for wild-type, hed1, dmc1 hed1, and dmc1 cells. At lest 50 nuclei were analyzed for each strain. C. Electron micrographs of SCs stained with silver. Both wild-type nuclei and one of the dmc1 hed1 nuclei have fully formed tripratite SCs.The hed1 dmc1 nucleus to the left displays regions of tripartite SC as well as unsynapsed regions. (TIFF) [file pgen.1003978.s006.tiff]

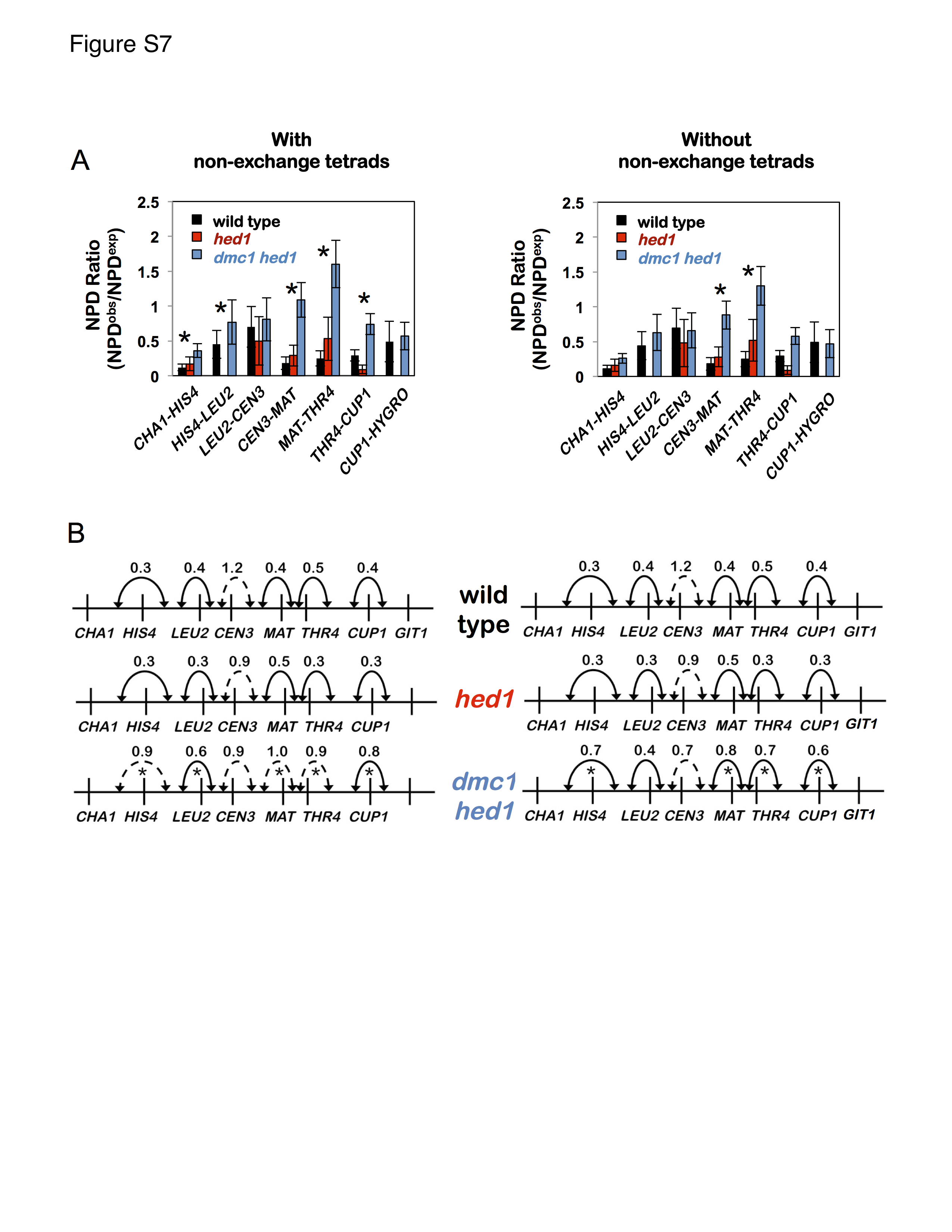

Supplement: Figure S7 — Analysis of crossover interference along chromosome III with and without non-exchange tetrads. A. NPD ratios. In the graph on the left, NPD ratio were calculated for the dmc1 hed1 dataset that included non-exchange tetrads. In the graph on the right, non-exchange tetrads were excluded from the dmc1 hed1 dataset. The asterisks indicate intervals in dmc1 hed1 dataset that show a significant change from wild type. B. Analysis of interference using the “adjacent intervals” approach of Malkova et al. [76]. Ratios of map distances AdjCO/AdjPD for wild-type, hed1, and dmc1 hed1 are shown. The reported ratios are averages of the two ratios for each interval pair. Solid lines represent significant interference and dashed lines represent non-significant deviations from a ratio of one. The panels on the left shows the analysis for dmc1 hed1 with non-exchange tetrads included and the panel on the right shows the analysis for dmc1 hed1 with non-exchange tetrads excluded. The asterisks indicate intervals with a significant change from wild type. (TIFF) [file pgen.1003978.s007.tiff]

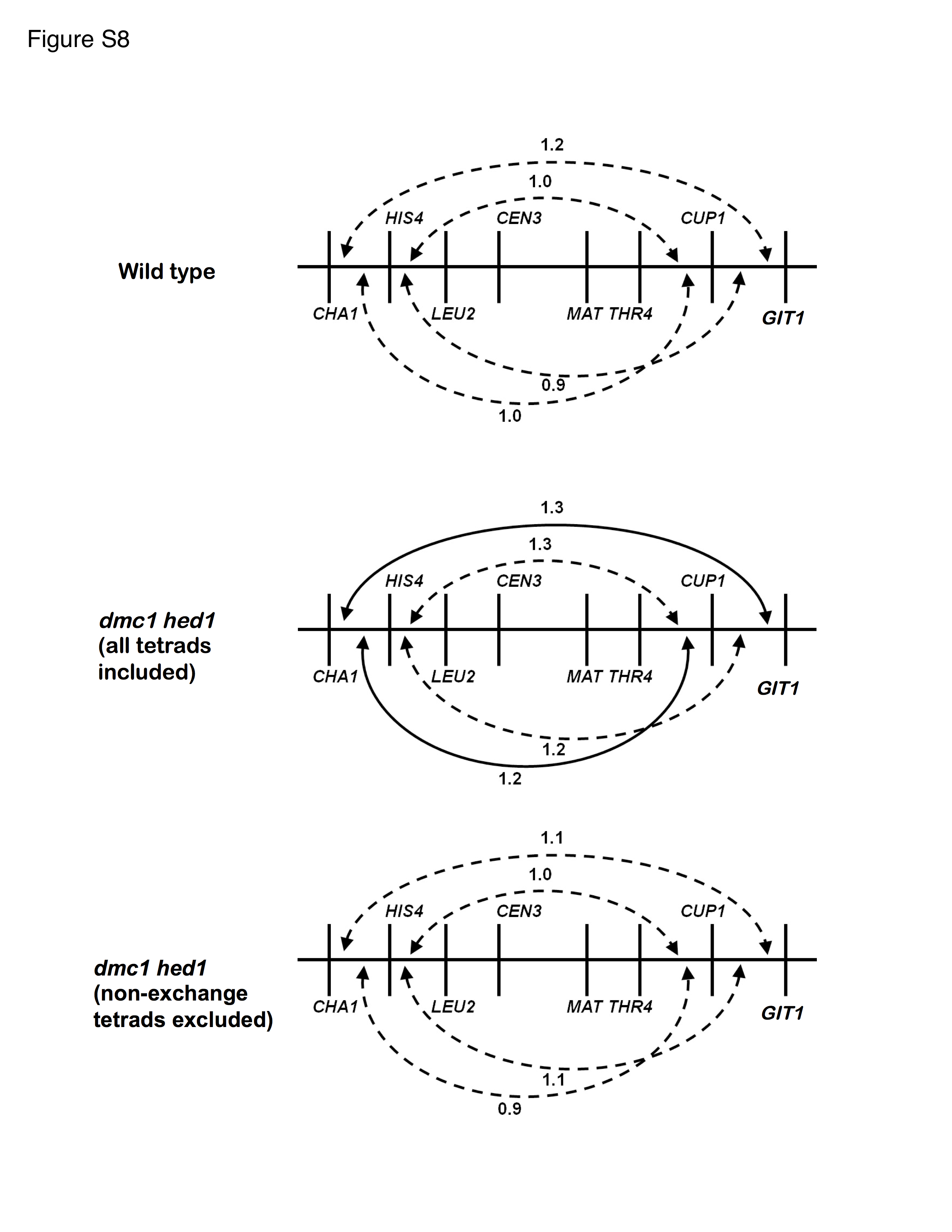

Supplement: Figure S8 — Analysis of crossover interference for distant intervals. Analysis of interference between distant intervals using the nearest neighbor approach of Malkova et al. (2004). Ratios of map distances AdjCO/AdjPD for wild type, dmc1 hed1 including non-exchange tetrads, and dmc1 hed1 excluding non-exchange tetrad. The number is the average of the two AdjCO/AdjPD ratios obtained by taking each interval as reference. Solid lines represent significant interference and dotted lines represent non-significant interference. dmc1 hed1 shows significant negative interference when non-exchange tetrads are included in the analysis. (TIFF) [file pgen.1003978.s008.tiff]
