# Supplementary material for: Meiotic Crossover Control by Concerted Action of Rad51-Dmc1 in Homolog Template Bias and Robust Homeostatic Regulation
Source: PLoS Genet. 2013 Dec 19;9(12):e1003978. doi: 10.1371/journal.pgen.1003978 (PMC3868528; doi:10.1371/journal.pgen.1003978)
Supplement: Table S1 — Quantitation of Red1, Zip1, and Zip3 localization. (PDF) [file pgen.1003978.s009.pdf]

Supplemental Table 1.
Quantitation of Red1, Zip1, and Zip3 localization.

	Strain			
	wild type	*hed1*	*dmc1 hed1*	*dmc1*
Density Red1 on Zip1	5 +/- 0.5	7 +/- 0.7 (0.041)*	2 +/- 0.5 (<0.0001)*	3 +/- 1.8
Density Red1 overall	9 +/- 0.6	11 +/- 0.6 (0.0001)*	8 +/- 0.9 (0.05)*	9 +/- 4
Density Red1 not associated with Zip1	3 +/- 0.4	4 +/- 0.7	5 +/- 0.8	5 +/- 2
Fraction Red1 on Zip1	62 +/- 5	59 +/- 4 (0.0751)	33 +/- 5 (<0.0001)*	38 +/- 12
Density Zip1	11 +/- 1	8 +/- 0.6 (<0.0001)*	5 +/- 0.6 (<0.0001)*	7 +/- 4
# Zip1 lines/cell (class 2)	5 +/- 0.7	4 +/- 0.7 (0.1)	3 +/- .3 (<0.0001)*	2 +/- 0.75
# Zip1 lines/cell (all)	6.5 +/- 0.84	5.7+/- 0.71 (0.22)	2.9 +/- 0.37 (<0.0001)*	1.7 +/- 0.75
# Zip3 foci on Zip1 lines	4 +/- 0.2	5 +/- 0.3 (0.0025)*	3 +/- 0.2 (<0.0001)*	3 +/- 0.5
# Zip3 foci	57 +/- 3	64 +/- 2 (<0.0001)*	45 +/- 1 (<0.0001)*	45 +/- 2 (<0.0001)*

All error is the 95% confidence interval. All P values are determined by the Mann-Whitney test. All P values marked with an asterisk are significant.
