# Supplementary material for: Meiotic Crossover Control by Concerted Action of Rad51-Dmc1 in Homolog Template Bias and Robust Homeostatic Regulation
Source: PLoS Genet. 2013 Dec 19;9(12):e1003978. doi: 10.1371/journal.pgen.1003978 (PMC3868528; doi:10.1371/journal.pgen.1003978)
Supplement: Table S2 — Quantitation and comparison of Red1 staining by STED microscopy. (PDF) [file pgen.1003978.s010.pdf]

Supplemental Table 2.
Quantitation and comparison of Red1 staining by STED microscopy.

	WT	*hed1*	*dmc1 hed1*
Density Red1 on Zip1	3 E-6+/- 0.6	5 E-6+/- 0.7 (0.0001)*	2E-6 +/- 0.5 (0.019)*
Density Red1 overall	5E-6 +/- 0.8	9 E-6+/- 1 (<0.0001)*	8E-6 +/- 2 (0.0003)*
Density Red1 not associated with Zip1	2E-6 +/- 4E-7	3E-06 +/- 7E-7	6E-6 +/- 1E-6
% Red1 on Zip1	64 +/- 6	63 +/- 5 (0.2)	24 +/- 4 (<0.0001)*

Measurements of the overall density of Red1 stain that colocalizes with Zip1 lines (arbitrarty units), overall density of Red1 (arbitrary units), and the percentage of Red1 density colocalizing with Zip1 lines over the total Red1 density. All P values were determined by the Mann-Whitney test. A P value of <0.05 is considered significant. All P values that are marked with an asterisk are significant. All error is the 95% confidence interval.
