# Supplementary material for: Meiotic Crossover Control by Concerted Action of Rad51-Dmc1 in Homolog Template Bias and Robust Homeostatic Regulation
Source: PLoS Genet. 2013 Dec 19;9(12):e1003978. doi: 10.1371/journal.pgen.1003978 (PMC3868528; doi:10.1371/journal.pgen.1003978)
Supplement: Table S3 — Analysis of crossover interference using non-parental ditype ratios. (PDF) [file pgen.1003978.s011.pdf]

Supplemental Table 3.
Analysis of crossover interference using non-parental ditype ratios.

		Interval						
		CHA1:HIS4	*HIS4:LEU2*	*LEU2:CEN3*	*CEN3:MAT*	*MAT:THR4*	*THR4:CUP1*	*CUP1:GIT1*
**wild type**	PD:NPD:TT	499:6:506	721:5:269	767:6:242	655:4:365	643:5:347	499:13:474	796:3:206
	NPD ratio	0.11+/-0.05	0.44+/-0.20	0.69+/-0.29	0.18+/-0.09	0.25+/-0.11	0.29+/-0.08	0.49+/-0.29
	P value	<0.0001*	0.0739	0.3896	0.0002*	0.0011*	<0.0001*	0.2174
hed1	PD:NPD:TT	259:3:216	342:0:130	358:2:114	280:4:195	334:3:132	235:2:229	389:0:84
	NPDobs/exp	0.16+/-0.09	0	0.48+/-0.34	0.28+/-0.14	0.52+/-0.30	0.09+/-0.06	0
	P value	0.0006*	0.019	0.3139	0.0105	0.2678	<0.0001*	0.1456
	**P value WT**	0.6272	0.0278	0.6394	0.5479	0.3981	0.0455	0.0911
dmc1 hed1	PD:NPD:TT	617:14:555	898:6:273	890:7:288	767:20:403	804:22:359	638:25:523	926:4:261
	NPDobs/exp	0.26+/-0.07	0.63+/-0.26	0.66+/-0.25	0.88+/-0.20	1.3+/-0.28	0.58+/-0.12	0.47+/-0.2
	P value	<0.0001*	0.2832	0.2981	0.615	0.3323	0.0133	0.139
	**P value WT**	0.0812	0.5624	0.9375	0.0014*	0.0005*	0.0443	0.9547

NPD ratios of NPDs observed/NPDs expected in the absence of interference. NPDs expected were calculated using the Papazian equation[75]. P values for interference were calculated by first calculating the fractions of PD_{exp}, NPD_{exp}, and TT_{exp} using the method of Stahl [74]. To determine the numbers of PD_{exp}, NPD_{exp}, and TT_{exp} the fractions expected were multiplied by the total number of tetrads. The numbers of PD_{exp}, NPD_{exp}, and TT_{exp} were then compared to the PD_{obs}, NPD_{obs}, and TT_{obs} using the chi-square goodness of fit test. All values were calculated using the Stahl Laboratory online tool: http://www.molbio.uoregon.edu/~fstahl/. PD, parental ditype; NPD, non-parental ditype; TT, tetratype. Non-exchange tetrads were excluded from this analysis. The P value WT rows are a P value for evaluating the difference between the NPD ratios of wild-type and the *hed1* single mutant and wild-type and the *dmc1 hed1* double mutant. The significance was determined by calculating a Z score: ((Ratio1-Ratio2)/(sqrt((SE1^2)+(SE2^2))) and corresponding P value for each comparison. The P value for significance using the Bonferroni Correction for 7 measurements is 0.007. All P values marked with an asterisk are significant.
