# Supplementary material for: Meiotic Crossover Control by Concerted Action of Rad51-Dmc1 in Homolog Template Bias and Robust Homeostatic Regulation
Source: PLoS Genet. 2013 Dec 19;9(12):e1003978. doi: 10.1371/journal.pgen.1003978 (PMC3868528; doi:10.1371/journal.pgen.1003978)
Supplement: Table S4 — Analysis of crossover interference using adjacent intervals. (PDF) [file pgen.1003978.s012.pdf]

Supplemental Table 4.
Analysis of crossover interference between adjacent intervals

			Reference Interval					
			CHA1:HIS4	*HIS4:LEU2*		*LEU2:CEN3*		*CEN3:MAT*
	Tetrad Class	Test Interval:	*HIS4:LEU2*	*CHA1:HIS4*	*LEU2:CEN3*	*HIS4:LEU2*	*CEN3:MAT*	*LEU:CEN3*
wild type	Adj PD	PD:NPD:TT	265:3:217	265:6:448	509:6:206	509:4:240	488:2:268	488:2:153
		cM	24	33	17	18	19	13
	AdjCO	PD:NPD:TT	454:2:52	220:0:54	244:0:29	212:1:28	155:2:91	270:4:89
		cM	6.3	9.9	5.3	7.0	21	16
		Ratio map distance	0.3	0.3	0.3	0.4	1.1	1.2
		P value	<0.0001*	<0.0001*	<0.0001*	<0.0001*	0.4656	0.2765
hed1	AdjPD	PD:NPD:TT	148:0:105	148:2:192	237:2:103	237:0:119	205:3:149	205:1:72
		cM	20	30	16	16	23	14
	AdjCO	PD:NPD:TT	194:0:25	105:1:24	119:0:11	105:0:11	73:1:42	152:1:42
		cM	5.7	12	4.2	4.7	21	12
		Ratio Map distance	0.3	0.4	0.3	0.3	0.9	0.9
		P value	<0.0001*	<0.0001*	<0.0001*	<0.0001*	0.6863	0.6461
		**P value WT**	0.9998	0.0172 *	0.9999	0.9999	0.0002*	<0.0001 *
dmc1 hed1	AdjPD	PD:NPD:TT	440:4:172	440:11:451	644:7:247	644:6:233	556:17:315	556:5:203
		cM	16	29	16	15	24	15
	AdjCO	PD:NPD:TT	462:2:101	176:3:100	239:0:40	254:0:40	208:2:83	332:2:83
		cM	10	21	7.2	6.8	16	11
		Ratio Map distance	0.6	0.7	0.4	0.4	0.7	0.7
		P value	0.0001*	0.0001*	<0.0001*	<0.0001*	0.0158	0.03151
		**P value WT**	<0.0001*	<0.0001*	0.4749	0.9999	0.0031*	0.0354*

Crossover interference was measured by the method of Malkova et al. [76]. For each reference interval, tetrads were divided into AdjCO (TTs and NPDs) and AdjPD (PDs). Distributions of tetrad types were then determined for the neighboring intervals and compared using the G test. Map distances were also calculated using the Perkins equation [101] using the Stahl laboratory online tool and expressed as a ratio (cM^{AdjCO}/cM^{AdjPD}). A ratio of <1 and P value of ≤0.004 indicates positive interference between the two intervals based on the Bonferroni Correction for 12 measurements. The ratio of the map distances in the test interval is taken as strength of interference. All P values marked with an asterisk indicate significant differences. All P values marked with an asterisk indicate significant differences. “P value WT” compares the strength of interference in the mutant situation to wild type by bootstrapping the wild-type data to create a new data set in which the sample size is equal to the sample size of the mutant data set. This process was repeated 10^6 times and map distance ratios were then determined for each recreated data set. The frequency with which the ratios from the recreated data sets matched the ratio obtained from the mutant data set was then determined. The P value is corrected for multiple testing. Non-exchange tetrads were excluded from this analysis.

Supplemental Table 4. Continued.
Analysis of crossover interference between adjacent intervals

			Reference Interval					
			CEN3:MAT	*MAT:THR4*		*THR4:CUP1*		*CUP1:GIT1*
	Tetrad Class	Test Interval:	*MAT:THR4*	*CEN3:MAT*	*THR4:CUP1*	*MAT:THR4*	*CUP1:GIT1*	*THR4:CUP1*
wild type	AdjPD	PD:NPD:TT	347:3:282	347:3:292	260:10:364	260:5:232	349:2:144	349:12:412
		cM	24	24	33	26	16	31
	AdjCO	PD:NPD:TT	297:2:65	285:1:66	237:3:103	374:0:106	424:1:56	146:1:56
		cM	11	10	18	11	6.4	15
		Ratio map distance	0.4	0.4	0.5	0.4	0.4	0.5
		P value	<0.0001*	<0.0001*	<0.0001*	<0.0001*	<0.0001*	<0.0001*
hed1	AdjPD	PD:NPD:TT	180:2:99	180:4:159	141:2:198	141:3:99	178:0:66	178:2:209
		cM	20	27	32	24	14	29
	AdjCO	PD:NPD:TT	163:1:33	101:0:34	102:0:31	200:0:31	211:0:17	66:0:17
		cM	9.9	13	12	6.7	3.7	10
		Ratio map distance	0.5	0.5	0.4	0.3	0.3	0.4
		P value	<0.0001*	<0.0001*	<0.0001*	<0.0001*	<0.0001*	<0.0001*
		**P value WT**	0.9999	0.9999	0.0249*	<0.0001*	0.0178*	0.0473
dmc1 hed1	AdjPD	PD:NPD:TT	496:14:259	496:13:304	394:17:394	394:17:227	457:3:179	457:17:446
		cM	22	24	31	26	15	30
	AdjCO	PD:NPD:TT	310:8:96	270:7:95	245:8:125	411:5:128	463:1:81	182:8:74
		cM	17	18	23	15	8.0	23
		Ratio map distance	0.8	0.8	0.7	0.6	0.5	0.8
		P value	0.0007*	0.0003*	<0.0001*	<0.0001*	<0.0001*	<0.0001*
		**P value WT**	0.0022*	<0.0001*	0.0425*	0.0555	0.8137	0.0158*
