# Supplementary material for: Meiotic Crossover Control by Concerted Action of Rad51-Dmc1 in Homolog Template Bias and Robust Homeostatic Regulation
Source: PLoS Genet. 2013 Dec 19;9(12):e1003978. doi: 10.1371/journal.pgen.1003978 (PMC3868528; doi:10.1371/journal.pgen.1003978)
Supplement: Table S5 — Analysis of crossover interference for distant intervals. (PDF) [file pgen.1003978.s013.pdf]

Supplemental Table 5.
Analysis of coincident crossovers involving distant intervals

			Reference Interval			
			CHA1:HIS4	*CUP1:GIT1*	*HIS4:LEU2*	*THR4:CUP1*
	Tetrad Class	Test Interval:	*CUP1:GIT1*	*CHA1:HIS4*	*THR4:CUP1*	*HIS4:LEU2*
WT	Adj PD	PD:NPD:TT	401:1:92	401:4:383	359:11:324	359:5:121
		cM	9.92	25.82	28.1	15.57
	AdjCO	PD:NPD:TT	387:2:109	93:2:109	126:2:134	335:0:136
		cM	12.15	29.66	27.86	14.44
		Ratio map distance	1.2	1.1	1.0	0.9
		P value	0.3941	0.2551	0.3827	0.03289
dmc1 hed1 (all tetrads included)	Adj PD	PD:NPD:TT	695:3:129	695:10:427	696:17:392	696:4:148
		cM	8.89	21.51	22.35	10.14
	AdjCO	PD:NPD:TT	437:1:129	132:4:126	152:8:118	409:2:124
		cM	11.9	28.63	29.86	12.71
		Ratio map distance	1.3	1.3	1.3	1.3
		P value	0.0016*	0.0030*	0.0214	0.0257
dmc1 hed1 (non-exchange tetrads excluded	AdjPD	PD:NPD:TT	486:3:129	486:10:427	487:17:392	487:4:148
		cM	11.89	26.38	27.57	13.47
	AdjCO	PD:NPD:TT	437:1:129	132:4:126	152:8:118	409:2:124
		cM	11.9	28.63	29.86	12.71
		Ratio Map distance	1.0	1.1	1.1	0.9
		P value	0.4955	0.6287	0.5551	0.8858

This analysis demonstrates that the total data set for the *dmc1 hed1* mutant displays the signature of negative interference for coincident COs involving unlinked intervals. Removing the non-exchange chromosomes from the analysis eliminates the signature of negative interference. This result suggests that the apparent negative interference in *dmc1 hed1* results from the contribution of non-exchange tetrads to the data. Therefore, the analysis provides evidence that most or all non-exchange tetrads represent a distinct subpopulation that is not engaged in crossover control. For each reference interval, tetrads were divided into AdjCO (TTs and NPDs) and AdjPD (PDs). Distributions of tetrad types were then determined for the test interval and compared using the G test. Map distances were also calculated using the Perkins equation [101] using the Stahl laboratory online tool and expressed as a ratio (cM^{AdjCO}/cM^{AdjPD}). A ratio of >1 and P value of ≤0.006 indicates negative interference between the two intervals based on the Bonferroni Correction for 12 measurements. The ratio of the map distances in the test interval is taken as strength of interference. All P values marked with an asterisk indicate significant differences.

Supplemental Table 5. Continued.
Analysis of coincident crossovers involving distant intervals

			Reference Interval			
			THR4:CUP1	*CHA1:HIS4*	*HIS4:LEU2*	*CUP1:GIT1*
	Tetrad Class	Test Interval:	*CHA1:HIS4*	*THR4:CUP1*	*CUP1:GIT1*	*HIS4:LEU2*
WT	Adj PD	PD:NPD:TT	249:3:245	249:8:227	558:3:148	558:4:213
		cM	26.46	28.41	11.71	15.29
	AdjCO	PD:NPD:TT	235:3:237	248:5:235	217:0:50	151:1:49
		cM	26.84	27.15	9.36	13.68
		Ratio map distance	1.0	1.0	0.8	0.9
		P value	0.9679	0.5387	0.5068	0.654
dmc1 hed1 (all tetrads included)	Adj PD	PD:NPD:TT	542:10:297	542:17:265	903:1:205	903:4:219
		cM	21.02	22.27	9.51	10.79
	AdjCO	PD:NPD:TT	282:4:254	307:8:250	223:3:53	206:2:54
		cM	25.47	26.37	12.72	12.6
		Ratio map distance	1.2	1.2	1.3	1.0
		P value	<0.001*	<0.001*	0.0473	0.4404
dmc1 hed1 (non-exchange tetrads excluded)	AdjPD	PD:NPD:TT	333:10:297	333:17:265	694:1:205	694:4:219
		cM	27.89	29.84	11.72	13.25
	AdjCO	PD:NPD:TT	282:4:254	307:8:250	223:3:53	206:2:54
		cM	25.74	26.37	12.72	12.6
		Ratio Map distance	0.9	0.9	1.1	1.0
		P value	0.4678	0.282	0.0275	0.3738
