# Supplementary material for: Meiotic Crossover Control by Concerted Action of Rad51-Dmc1 in Homolog Template Bias and Robust Homeostatic Regulation
Source: PLoS Genet. 2013 Dec 19;9(12):e1003978. doi: 10.1371/journal.pgen.1003978 (PMC3868528; doi:10.1371/journal.pgen.1003978)
Supplement: Table S6 — Yeast strains used in this study. (PDF) [file pgen.1003978.s014.pdf]

Supplemental Table 6. Yeast strains used in this study (all in the SK1 strain background)	
**Strain**	**Genotype**
NHY 1804	*ho::hisG/" leu2::hisG/" ura3(ΔSma-Pst)/" HIS4::LEU2-(BamHI; +ori)/his4-X::LEU2-(NgoMIV; +ori)-URA3*
NHY 3124	*ho::hisG/" leu2::hisG/" ura3(ΔSma-Pst)/' HIS4::LEU2-(BamHI; +ori)/his4-X::LEU2-(NgoMIV; +ori)--URA3 hed1Δ::kanMX/"*
NHY 4096	*ho::hisG/" leu2::hisG/" ura3(ΔSma-Pst)/" HIS4::LEU2- (BamHI; + ori)/X::LEU2- (NgoMIV; + ori)--URA3 dmc1Δ::kanMX4/"*
NHY 3157	*ho::hisG/" leu2::hisG/" ura3(ΔSma-Pst)/" HIS4::LEU2-(BamHI; +ori)/his4-X::LEU2-(NgoMIV; +ori)--URA3 hed1Δ::kanMX/" dmc1Δ::kanMX4/"*
NHY 2328 X NHY 2498	*ho::hisG/" ura3(ΔSma-Pst)/" Δade2/" thr4-B/THR4 lys2/" trp1Δ/trp1Δ cup1Δ/cup1Δ TRP1--on chr3/CUP1--on chr3 leu2-r/LEU2 GAL3+/" CEN3::URA3/CEN3::LYS2 can1-r/CAN1 CYH1/cyh-r CHA1/cha1::ADE2 GIT1::hphMX4*
NHY 3388 X NHY 3390	*ho::hisG/" ura3(ΔSma-Pst)/" Δade2/" thr4-B/THR4 lys2/" trp1Δ/trp1Δ cup1Δ/cup1Δ TRP1--on chr3/CUP1--on chr3 leu2-r/LEU2 GAL3+/" CEN3::URA3/CEN3::LYS2 can1-r/CAN1 CYH1/cyh-r CHA1/cha1::ADE2 GIT1::hphMX4 hed1Δ::kanMX4/"*
NHY 4488 X NHY 4489	*ho::hisG/" ura3(ΔSma-Pst)/" Δade2/" thr4-B/THR4 lys2/" trp1Δ/trp1Δ cup1Δ/cup1Δ TRP1--on chr3/CUP1--on chr3 leu2-r/LEU2 GAL3+/" CEN3::URA3/CEN3::LYS2 can1-r/CAN1 CYH1/cyh-r CHA1/cha1::ADE2 GIT1::hphMX4 hed1Δ::kanMX4/" dmc1Δ::kanMX4/"*
NHY 4042	*ho::hisG/" leu2::hisG/" ura3(ΔSma-Pst)/" HIS4::LEU2-(BamHI; +ori)/his4-X::LEU2-(NgoMIV; +ori)--URA3 mnd1Δ::kanMX4/"*
NHY 4053	*ho::hisG/" leu2::hisG/" ura3(ΔSma-Pst)/" HIS4::LEU2-(BamHI; +ori)/his4-X::LEU2-(NgoMIV; +ori)--URA3 mnd1Δ::kanMX4/" hed1Δ::kanMX4/"*
NHY 4181	*ho::hisG/" leu2::hisG/" ura3(ΔSma-Pst)/" HIS4::LEU2-(BamHI; +ori)/his4-X::LEU2-(NgoMIV; +ori)--URA3 hed1Δ::kanMX/" dmc1Δ::kanMX4/" mnd1Δ::kanMX4/"*
NHY 1736	*ho::hisG/" leu2::hisG/" ura3(ΔSma-Pst)/" HIS4::LEU2- (BamHI; + ori)/his4-X::LEU2- (NgoMIV; + ori)--URA3 Δrad51::hisG/"*
NHY 3559	*ho::hisG/" leu2::hisG/" ura3(ΔSma-Pst)/" HIS4::LEU2-(BamHI; +ori)/his4-X::LEU2-(NgoMIV; +ori)--URA3 hed1Δ::kanMX/" Δrad51::hisG/"*
NHY 3520	*ho::hisG/" leu2::hisG/" ura3(ΔSma-Pst)/" HIS4::LEU2-(BamHI; +ori)/his4-X::LEU2-(NgoMIV; +ori)--URA3 dmc1Δ::kanMX4/" Δrad51::hisG/"*
NHY 3519	*ho::hisG/" leu2::hisG/" ura3(ΔSma-Pst)/" HIS4::LEU2-(BamHI; +ori)/his4-X::LEU2-(NgoMIV; +ori)--URA3 hed1Δ::kanMX/" dmc1Δ::kanMX4/" Δrad51::hisG/"*
NHY 3944	*ho::hisG/" leu2::hisG/" ura3(ΔSma-Pst)/" HIS4::LEU2-(BamHI; +ori)/his4-X::LEU2-(NgoMIV; +ori)--URA3 mei5Δ::kanMX4/"*
NHY 4031	*ho::hisG/" leu2::hisG/" ura3(ΔSma-Pst)/" HIS4::LEU2-(BamHI; +ori)/his4-X::LEU2-(NgoMIV; +ori)--URA3 mei5Δ::kanMX4/" hed1Δ::kanMX4/"*
NHY 4763	*hoΔ/" leu2/" ura3/" ERG1-(Sal1)/ERG1-(SpeI), HIS4::LEU2-(BamHI; +ori)/his4-X::LEU2-(NgoMIV; +ori)--URA*
NHY 4765	*hoΔ/" leu2/" ura3/" ERG1-(Sal1)/ERG1-(SpeI), HIS4::LEU2-(BamHI; +ori)/his4-X::LEU2-(NgoMIV; +ori)--URA dmc1Δ::KanMX4/" hed1Δ::KanMX4*
DKB 1772	*ho::hisG/", HIS4::LEU2-(NBam)/his4X::LEU2-(NBam)-URA3, leu2::hisG/", ura3 (ΔPst-Sma)/"*
DKB 2904	*ho::hisG/", lys2/", leu2::hisG/", dmc1Δ::ARG4/", HIS4::LEU2-(NBam)/his4X::LEU2-(NBam)-URA3, ura3/ura3 or URA, ARG4/"*
DKB 2905	*ho::hisG/", lys2/", leu2::hisG/", HIS4::LEU2-(NBam)/his4X::LEU2-(NBam)-URA3, ura3/", ARG4/", trp/", hed1::KanMX4/"*
DKB 2908	*ho::hisG/", lys2/", leu2::hisG/", dmc1Δ::ARG4/", HIS4::LEU2-(NBam)/his4X::LEU2-(NBam)-URA3, ura3/", ARG4/", hed1::KanMX4/", trp/"*
DKB 3348	*ho::hisG/",HIS4::LEU2 -(NBam)/his4X::LEU2-(NBam)-URA3, leu2::hisG/", ura3(ΔPst-Sma/", mek1-as1/", hed1::KanMX4/", trp/"*
DKB 3343	*ho::hisG/",leu2::hisG/",HIS4::LEU2-(NBam)/his4x::LEU2-(NBam)-URA3, ura3(ΔPst-Sma)/",mek1-as1/", lys2/LYS2*
DKB 3497	*ho::hisG/", his4X::LEU2-(NgoMIV+ori)-URA3/HIS4::LEU2-(BamHI; +ori), leu2::hisG/", ura3 (ΔPst-Sma)/" , mek1Δ::NAT/"*
DKB 3512	*ho::hisG/", his4X::LEU2-(NgoMIV+ ori)-URA3/HIS4X::LEU2-(BamHI; +ori)-ura3, leu2::hisG/", ura3 (ΔPst-Sma) /", mek1Δ::NAT/", hed1::KANMX4*
DKB 3048	*ho::hisG/", lys2/", ura3/", leu2::hisG/", his4X::LEU2-(NBam)-URA3/HIS4::LEU2-(NBam), rad51Δ::hisG/", arg-nsp/"*
DKB 3050	*ho::hisG/ho::LYS2, lys2/", ura3/", leu2::hisG/", his4X::LEU2-(NBam)-URA3/HIS4::LEU2-(NBam), trp/", hed1::KanMX4/", rad51Δ::hisG/", arg-nsp/"*
