# Supplementary material for: Meiotic Crossover Control by Concerted Action of Rad51-Dmc1 in Homolog Template Bias and Robust Homeostatic Regulation
Source: PLoS Genet. 2013 Dec 19;9(12):e1003978. doi: 10.1371/journal.pgen.1003978 (PMC3868528; doi:10.1371/journal.pgen.1003978)
Supplement: Text S1 — Supplementary discussion. (PDF) [file pgen.1003978.s015.pdf]

Supplementary Discussion

Additional rationale for purging data sets of non-exchange tetrads prior to interference analysis: analysis of coincident crossovers in distant intervals.

The tetrad data obtained from the *dmc1 hed1* mutant strain contained a substantial number of non-exchange chromosomes (E0). As mentioned in the text, the Poisson distribution predicts that, if the distribution of crossovers between chromosome III is random and there are an average of 2.3 crossovers per meiosis, the fraction of tetrads predicted to have 0 crossovers is 10%. However, we observed that 15% of *dmc1 hed1* tetrads are E0. This finding indicates that it is not possible to account for all E0 tetrads as resulting from stochastic failure of crossing over. Instead, the data suggest that a fraction of cells fails to meet some precondition for crossing over between chromosome III. The question arises, what is the true fraction of cells that fails to engage in crossing over?

To shed light on the fraction of chromosomes that fails to engage in crossing over, we examined the impact of E0 tetrads on cM^{AdjCO}/cM^{AdjPD} map distance ratios for unlinked test intervals that show no evidence of interference in wild-type tetrads i.e. the cM^{AdjCO}/cM^{AdjPD} map distance ratios are not different from 1 (Figure S8, Table S5). In data sets containing a substantial fraction of E0 tetrads, such as the *dmc1 hed1* dataset, elimination of E0s will decrease cM^{AdjCO}/cM^{AdjPD} map distance ratios. This decrease is accounted for as follows. First, the map distance calculated for a test interval in the AdjCO category will be unchanged by E0 purging, because, by definition, AdjCO tetrads do not include E0 chromosomes. Second, elimination of E0s from the AdjPD category will increase map distance because the size of the PD tetrad class will be decreased and PDs only contribute to the denominator of the mapping equation ([(T+6NDP)/2(T+NPD+PD)]x100). Thus, purging of E0s will decrease

the cM^{AdjCO}/cM^{AdjPD} map distance ratio by increasing the map distance calculated from AdjPD tetrads (cM^{AdjPD}).

We considered two models for the source of E0s:

(i) All E0 tetrads reflect a distinct subpopulation that does not participate in crossing over. In this case, the cM^{AdjCO}/cM^{AdjPD} ratios for unlinked intervals calculated using the complete dataset are expected to be >1, because of specific reductions of the AdjPD map distance caused by the E0 class. Further, purging the dataset of E0s is expected to generate ratios for distant test intervals that do not differ from 1. For the *dmc1 hed1* data set, the predictions of this model were fulfilled; cM^{AdjCO}/cM^{AdjPD} ratios were significantly greater than 1 for two of four intervals tested when the full data set was analyzed, but none of the ratios differed significantly from 1 when the data set was purged of E0s (Figure S8, Table S5).

(ii) Only 1/3 of E0 tetrads (5% of total) arise because of failure to meet a precondition required for crossover formation. The remaining 2/3rds result from a random distribution of crossovers among the crossover-competent population of cells (95% of total). If this were the case, purging of all E0's might result in cM^{AdjCO}/cM^{AdjPD} ratios for distant intervals that are significantly less than one, i.e. purging all E0s might artificially enhance of positive interference. Contrary to this prediction, cM^{AdjCO}/cM^{AdjPD} ratios for distant intervals showed no evidence of positive interference (ratios <1) when the data were purged of all E0's.

We view these results as providing support for our conclusion that most or all E0 tetrads in the *dmc1 hed1* dataset represent a distinct subset of cells that fail to engage in crossover recombination. The ≥16-fold decrease in gene conversions seen in non-exchange tetrads provides independent evidence supporting this view. The results of this analysis also show that inclusion of E0s erroneously diminishes the signature of positive crossover interference.

Thus, analysis of data purged of E0s provides the most meaningful approach to determine the strength of crossover interference in "crossover competent" *dmc1 hed1* cells.

B. Up-regulation of crossover bias does not fully account for the efficient crossover compensation observed in *dmc1 hed1* cells. At each of two recombination hotspots *HIS4::LEU2* and *ERG1*, the fraction of inter-homolog joint molecules is reduced 5-fold in *dmc1 hed1*. However final crossover levels are only reduced 3.5- and 2-fold, respectively (Figure 9B, Figure 2G), indicating that *dmc1 hed1* cells partially compensate for the defect in homolog bias. Analysis of crossover/non-crossover ratios indicates that increasing the crossover fraction is one mechanism that compensates for reduced homolog bias (Figure 7C, Figure 7F). However, the observed increases in crossover/non-crossover ratios do not fully account for the levels of crossovers observed in *dmc1 hed1*. This conclusion is based on calculations that follow:

At *HIS4::LEU2*, the ratio of crossovers to non-crossovers is 1.4 in wild-type and 1.8 in *dmc1 hed1*. By algebraic conversion, 58% of recombination events are crossovers in wild-type and 64% of events are crossovers in *dmc1 hed1*, an increase of 1.1-fold or 10%. However, the total observed compensation for the 5-fold reduction in interhomolog joint molecules is greater than 10%. Interhomolog joint molecule levels in *dmc1 hed1* are 20% of those in wild-type, whereas final crossover levels are 29% of those in wild-type, a 1.45-fold (0.29/0.20) or 45% compensation. Thus, the increase in crossover yield afforded by enhancement of the fraction of events that form crossovers in *dmc1 hed1* only accounts for about 22% (0.10/0.45) of the total observed compensation.

At *ERG1*, the ratio of crossovers to non-crossovers is 1.0 in wild-type and 4.9 in *dmc1 hed1*, corresponding to crossover fractions of 50% and 83% respectively. Thus, compensation by

enhancement of crossover bias is 1.7-fold (0.83/0.5) or a 70% increase. Given that interhomolog joints are reduced to 20% of wild-type levels while crossovers are reduced to 50%, the total compensation is 2.5-fold or a 150% increase relative to the level predicted from joint molecule abundance. Thus, the enhanced crossover fraction accounts for about 47% (0.7/1.50) of the observed compensation for the homolog bias defect at *ERG1*.

In summary, enhanced crossover bias does not fully account for the observed compensation for the primary recombination defect in *dmc1 hed1* cells. We propose in the main text that a second compensatory mechanism, most likely involving an increase in the number of DSBs, acts in *dmc1 hed1* to compensate for the primary recombination defect.

Note: Our observation that homeostatic compensation at the *HIS4::LEU2* hotspot is relatively inefficient compared to other loci is consistent with previous analysis [18] and suggests that this artificially generated site may not be as efficiently regulated as natural hotspots.
